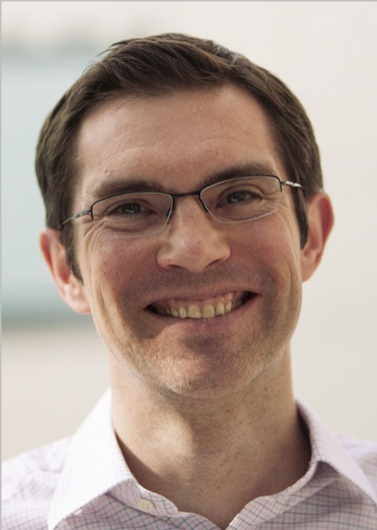# The 2‐Pyridyl Problem: Challenging Nucleophiles in Cross‐Coupling Arylations

**DOI:** 10.1002/anie.202010631

**Published:** 2020-11-17

**Authors:** Xinlan A. F. Cook, Antoine de Gombert, Janette McKnight, Loïc R. E. Pantaine, Michael C. Willis

**Affiliations:** ^1^ Chemistry Research Laboratory Oxford University 12 Mansfield Road Oxford OX1 3TA UK

**Keywords:** biaryl, catalysis, palladium, pyridine, synthetic methods

## Abstract

Azine‐containing biaryls are ubiquitous scaffolds in many areas of chemistry, and efficient methods for their synthesis are continually desired. Pyridine rings are prominent amongst these motifs. Transition‐metal‐catalysed cross‐coupling reactions have been widely used for their synthesis and functionalisation as they often provide a swift and tuneable route to related biaryl scaffolds. However, 2‐pyridine organometallics are capricious coupling partners and 2‐pyridyl boron reagents in particular are notorious for their instability and poor reactivity in Suzuki–Miyaura cross‐coupling reactions. The synthesis of pyridine‐containing biaryls is therefore limited, and methods for the formation of unsymmetrical 2,2′‐bis‐pyridines are scarce. This Review focuses on the methods developed for the challenging coupling of 2‐pyridine nucleophiles with (hetero)aryl electrophiles, and ranges from traditional cross‐coupling processes to alternative nucleophilic reagents and novel main group approaches.

## Graphical abstract

This Review focuses on methods developed as solutions for the challenging coupling of 2‐pyridyl nucleophiles with (hetero)aryl electrophiles. The topic encompasses traditional cross‐coupling processes, modifications to the Suzuki–Miyaura coupling, as well as alternative nucleophilic reagents and recent developments in C−H activation.

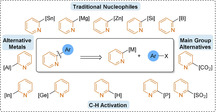

## 1 Introduction

**Figure 1:**
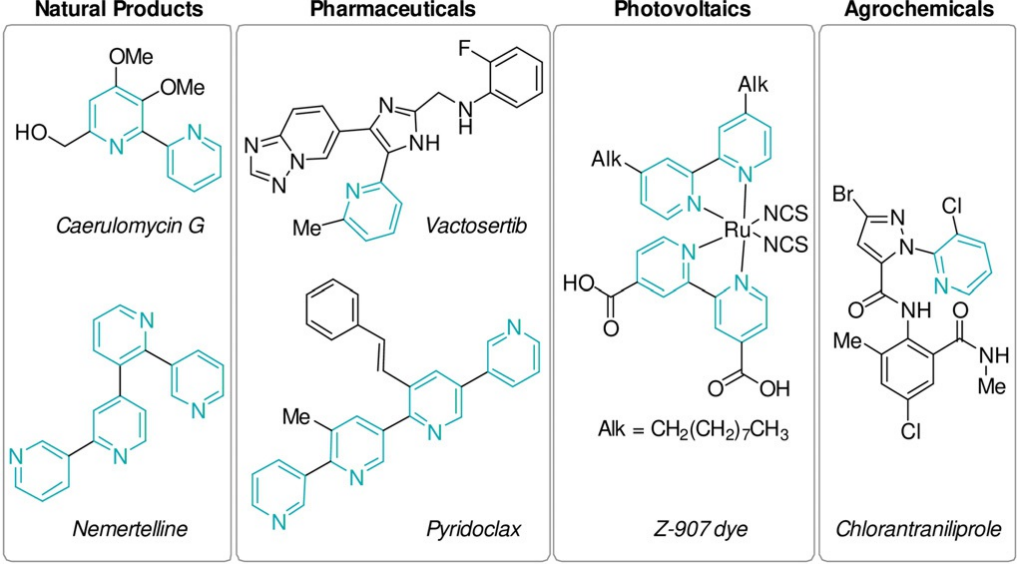
Pyridine‐derivatives across various applications.

Pyridine derivatives are a common structural motif in natural products^[1]^ and have found applications in diverse fields, from functional materials (photovoltaics, ligands, dyes),^[2]^ to agrochemistry^[3]^ and medicinal chemistry (Figure 1).^[4]^ The pyridine core ranks second out of the most used heterocycles in medicinal compounds,^[4a]^ and bipyridines are frequently employed as ligands in coordination chemistry.^[5]^ A historical review of the first fifty years of the chemistry of 2,2′‐bipyridines was recently reported by Housecroft and Constable.^[6]^

**Scheme 1:**
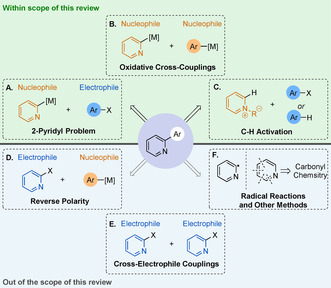
Synthesis of 2‐pyridine‐containing biaryls: scope of this Review.

Despite the clear importance of pyridines, their functionalisation remains challenging, particularly at the 2‐position. In transition‐metal‐catalysed coupling chemistry, the use of 2‐pyridyl derivatives as nucleophilic coupling partners has proved notoriously difficult in traditional cross‐coupling reactions. This challenge was coined the “2‐pyridyl organometallic cross‐coupling problem” by Fagnou and co‐workers in 2005 (Scheme 1 A).^[7]^ Considering the popularity of the Suzuki–Miyaura cross‐coupling (SMC), there is particular interest in improving the poor reaction success of 2‐pyridyl boron nucleophiles. For example, the challenges of coupling 2‐pyridyl boronates lead Rault and co‐workers to tune their synthetic pathway to avoid such species while preparing Nemertelline.^[8]^ The extent of the 2‐pyridyl problem is highlighted by the results of a survey of the use of 2‐pyridyl boron reagents in Suzuki chemistry, taken from the Pfizer internal electronic laboratory notebook: less than 8 % of the reactions surveyed obtained a product yield of at least 20 %.^[9]^ Oxidative cross‐couplings of two pyridyl nucleophiles are evidently underdeveloped for the same reasons (Scheme 1 B). Given these challenges, it follows that many innovative developments have emerged, and these are the focus of this Review. A note on terminology; although cross‐coupling reactions are not traditional nucleophile+ electrophile combinations, for pragmatic reasons, in this Review we will refer to aryl−metal species as the nucleophilic fragment, while aryl halides (or pseudohalides) will be referred to as the electrophile.

The 2‐pyridyl problem can be circumvented by the formal inversion of polarity of the coupling partners (Scheme 1 D). 2‐Halopyridines are excellent electrophilic partners, compatible with a range of cross‐coupling conditions. Indeed, experimental^[10]^ and theoretical^[11]^ results show that 2,3‐ and 2,4‐dihalopyridines react regioselectively at the position adjacent to the nitrogen, where the C−X bond has a lower bond dissociation energy. However, such an approach is less attractive in discovery chemistry as it fails to exploit the vast libraries of commercially available halogen‐substituted arenes. Furthermore, this reverse‐polarity approach is not compatible with the preparation of non‐symmetrical 2,2′‐bipyridines and other 2,2′‐bis‐azine‐linked derivatives.

The cross‐coupling of two electrophiles, derived from the classical Ullmann reaction,^[12]^ also offers an alternative to the 2‐pyridyl problem (Scheme 1 E). Since its first application to the synthesis of bipyridine in 1928,^[13]^ numerous metal‐catalysed Ullmann‐type homocouplings have been developed for the synthesis of symmetrical 2,2′‐bipyridines and bis‐azine derivatives.^[14]^ However, the reductive coupling of two different electrophiles remains difficult owing to selectivity issues. Cross‐electrophile couplings leading to non‐symmetric biheteroaryl compounds remain underdeveloped and do not yet represent a general solution to the 2‐pyridyl problem.^[15]^ Direct arylation through palladium‐catalysed C−H activation has emerged as an attractive alternative to classic cross‐coupling reactions,^[16]^ especially for heterocycles as the presence of the heteroatom activates a specific C−H bond, increasing reactivity and regioselectivity.^[17]^ Significant progress in this field has recently been made concerning the direct arylation of 2‐pyridine derivatives (Scheme 1 C).^[18]^ Functionalised pyridines can also be obtained de novo, using carbonyl fragments (Scheme 1 F). However, this Review focuses on cross‐coupling processes, which provide a swift and tuneable route to a broad range of scaffolds. Reactions involving 2‐pyridyl radical intermediates are not discussed in this Review.

Rather than discussing a broad range of heteroaromatic nucleophiles,[^19^, ^20^] we have chosen to focus on 2‐pyridyl nucleophiles as benchmark substrates. Indeed, 2‐pyridines are of prime importance and are notoriously one of the most challenging nucleophiles in cross‐coupling reactions. Focusing on other heterocycles,^[21]^ which are traditionally better performing nucleophiles, can be misleading when making the appropriate choice of reagents and conditions for more challenging substrates.

This Review aims to provide a critical overview of the progress that has been made towards a general solution to the 2‐pyridyl problem, ranging from traditional cross‐coupling arylations to more recent developments. The sections of this Review are organised by nucleophile type. This discussion of innovative strategies developed for various 2‐pyridyl nucleophiles should provide chemists with a set of resources and conditions applicable to a range of challenging heteroaromatic substrates.

## 2 Traditional Nucleophiles

### 2.1 2‐Pyridylzinc (Zn)

**Scheme 2:**
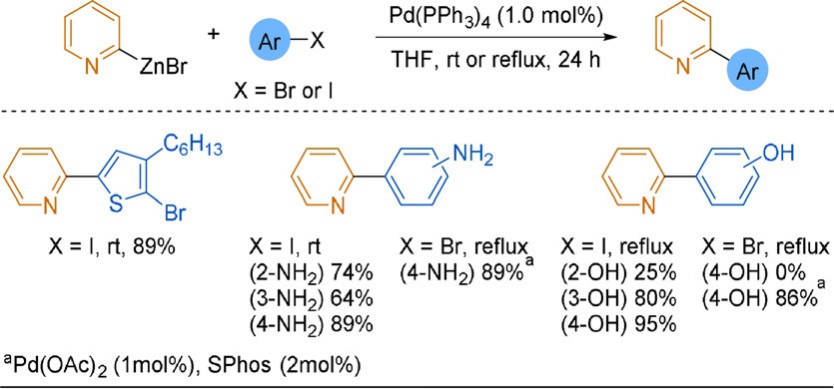
Selected examples of Negishi cross‐couplings catalysed by Pd(PPh_3_)_4_. rt=room temperature; THF=tetrahydrofuran; SPhos=2‐dicyclohexylphosphino‐2′,6′‐dimethoxybiphenyl.

Organozinc reagents can be obtained via direct, or transition‐metal‐catalysed, oxidative addition of zinc into carbon−halide bonds, transmetalation of metalated substrates with a zinc source such as ZnCl_2_ or ZnBr_2_, or by direct zincation of C−H bonds.^[22]^ These methods can be applied on multi‐kilogram scale to 2‐pyridyl substrates, which do not suffer from any particular instability compared to their carbocyclic analogues.^[23]^ Negishi cross‐coupling protocols developed for carbocyclic substrates^[24]^ were adapted to 2‐pyridyl derivatives without major changes.^[25]^ The relatively inexpensive catalyst Pd(PPh_3_)_4_ can be employed for the coupling of structurally simple 2‐pyridylzinc reagents with a wide variety of electrophiles, tolerating halides, amines and alcohols (Scheme 2).^[26]^

**Scheme 3:**
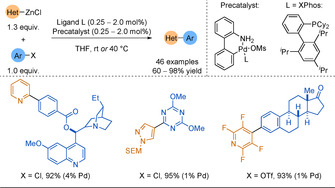
Use of XPhos Pd G3‐amido precatalyst for Negishi couplings under mild reaction conditions.

Hapke and Lützen showed that Pd(P^*t*^Bu_3_)_2_ could be used to form 5‐substituted 2,2′‐bipyridines (9 examples, 5–90 % yield).^[27]^ XPhos was subsequently found to be a more efficient ligand^[28]^ and was employed by Knochel, Buchwald and co‐workers for coupling 2‐pyridylzinc pivalate reagents.^[29]^ By employing zinc pivalate as the zinc source, these substrates could be weighed under air with minimal loss of activity. A range of functional groups such as esters, ketones, amides, anilines or nitriles were tolerated on the electrophile, but the pyridine core remained poorly functionalised (14 examples, 60–98 % yield). The Buchwald group also demonstrated that the use of their XPhos Pd G3‐amido precatalyst (Scheme 3) provided much improved activity in Negishi cross‐couplings.^[30]^ Although the coupling was only applied to unsubstituted 2‐pyridylzinc chloride, these mild reaction conditions allowed the coupling of a large scope of challenging nucleophiles, such as 5‐membered heterocycles bearing more than one heteroatom, or polyfluoro(hetero)aryl zinc reagents.

Organ and co‐workers showed the remarkable efficiency of a N‐heterocyclic carbene (NHC) ligand for the synthesis of sterically demanding tetra‐*ortho*‐substituted biaryls.^[31]^ They demonstrated that this catalyst was also efficient for a variety of heterocyclic substrates, including 2‐pyridylzinc bromide.

**Scheme 4:**
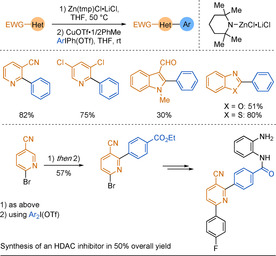
Copper‐catalysed coupling of electron‐deficient (hetero)arenes via direct zincation. tmp=2,2,6,6‐tetramethylpiperidine. EWG=electron withdrawing group.

More recently, Liu and Wang developed a direct coupling of electron‐deficient (hetero)arenes using iodonium salts.^[32]^ The base Zn(tmp)Cl⋅LiCl was selected to promote selective zincation. Subsequent coupling with iodonium salts under copper catalysis allowed a diverse scope of nucleophiles to be used (Scheme 4). No desired product was observed when the iodonium electrophile was replaced by its triflate or iodide equivalent. The synthetic utility of this method was illustrated by a rapid synthesis of a histone deacetylase inhibitor in 50 % overall yield from commercial 6‐bromonicotinonitrile.

### 2.2 2‐Pyridylstannanes (Sn)

2‐Pyridylstannanes usually provide robust, scalable, and high yielding reactions with aryl halides.[^21^, ^33^] 2‐(Tributylstannyl)pyridine is commercially available, and 2‐(trialkylstannyl)pyridyl derivatives can be obtained directly from 2‐bromopyridines using Sn_2_Bu_6_ in a palladium‐catalysed process,^[34]^ or via lithium/bromide exchange followed by quenching with trialkyltin chloride.^[35]^ However, recent reports of tin‐based cross‐coupling methodologies involving 2‐pyridyl substrates are scarce.^[36]^ The high toxicity of organotin compounds, the difficulty associated with the removal of tin impurities from reaction mixtures, and the low tolerance of tin residues in biological assays explain the reduced focus in this area. Nevertheless, it is important to mention that 2‐pyridylstannanes have been extensively used for the synthesis of nitrogen abundant molecules such as polypyridines,^[37]^ complex polyazine molecules,^[38]^ *tert*‐pyridines,^[39]^ and also medicinally relevant scaffolds such as analogues of the antitumour antibiotic lavandamycin^[40]^ as well as natural products.^[41]^

### 2.3 2‐Pyridyl Grignard Reagents (Mg)

**Scheme 5:**
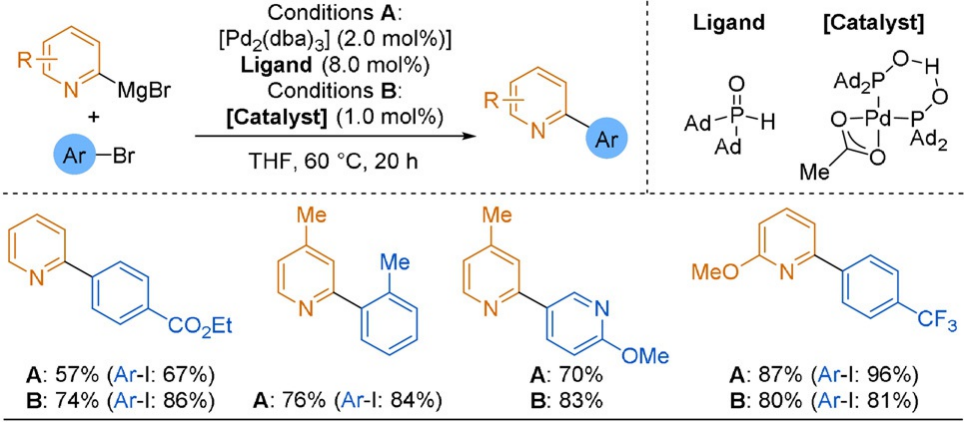
SPO ligands enabling the Kumada coupling of 2‐pyridyl Grignard reagents.

Although 2‐pyridyl Grignard reagents have long been known and easily accessed via magnesium/halide exchange reactions,^[42]^ their use in cross‐coupling reactions has remained limited.^[43]^ In 1982, Kumada, Suzuki and co‐workers reported a NiCl_2_(dppp)‐catalysed coupling of heterocyclic Grignard reagents with a range of heteroaromatic aryl halides.^[44]^ However, the formation of 2,2′‐bipyridine only proceeded in 13 % yield. In 2010, Ackermann, Schulzke and co‐workers showed the unique ability of secondary phosphine oxides (SPOs) to promote the palladium‐catalysed coupling of 2‐pyridyl Grignard reagents with aryl halides.^[45]^ In contrast, commonly employed phosphine and NHC ligands showed no or poor reactivity. Catalyst loading could be lowered to 1 mol % [Pd] using a preformed catalyst (Scheme 5, Conditions B) but the combination of Pd_2_(dba)_3_ and phosphine oxide ligand (1‐Ad)_2_P(O)H was also successful (Conditions A). 2‐Pyridylmagnesium bromide reacted in good to excellent yields with aromatic or heteroaromatic electrophiles (52–94 % yield), and the pyridine nucleophile could be substituted at positions 4 or 6 without loss of reactivity. However, functional group tolerance remains limited.

**Scheme 6:**
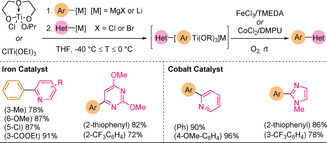
Sequential assembly with two Grignard reagents, titanium as the coupling agent and under Fe or Co catalysis. TMEDA=tetramethylethylenediamine; DMPU=*N*,*N*′‐dimethylpropyleneurea.

Duan and co‐workers reported the successful use of 2‐pyridyl Grignard reagents in the iron‐^[46]^ or cobalt‐mediated^[47]^ oxidative assembly of two aryl metal reagents using oxygen as an oxidant. The two arylmetal reagents were assembled sequentially to form a titanate complex [HetAr(ArTi(OR)_3_)M], and the reductive coupling was triggered by the addition of the iron or cobalt catalyst mixture under an oxygen atmosphere (Scheme 6). Both iron and cobalt protocols tolerated a range of aromatic and heteroaromatic substrates, but 2,2′‐bis‐azine‐linked derivatives could not be obtained.

### 2.4 Couplings with Alternative Metal Reagents

**Scheme 7:**
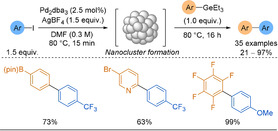
Selected scope examples, featuring sensitive functionality, of organogermane cross‐couplings to aryl iodides. dba=dibenzylideneacetone.

In 2019, Schoenebeck demonstrated that novel organogermanes could provide a solution to the problem posed by unstable 2‐pyridyl and polyfluoroaryl boronic acids in Suzuki reactions.^[48]^ Key benefits identified in this work: arylgermanes have low toxicity,^[49]^ were easily synthesised from triethylgermanium chloride using Grignard reagents, and demonstrated high stability to both acid and base. The reaction coupled aryl iodides or iodoniums chemoselectively to a variety of aryl and heteroaryl germanes (Scheme 7). Yields were noticeably lower for the heteroaryl germanes compared to the carbocyclic variants. Also, no heteroaryl electrophiles were coupled. Pentafluorophenyl germane coupled in excellent yields, highlighting the importance of this work for what would otherwise be a challenging SMC. Notably, under conventional palladium catalysis the organogermanes were unreactive, yet under Pd nanoparticle catalysis, with much lower catalyst loadings, high reactivity was shown. This, coupled with the chemoselectivity for iodo electrophiles, lends well to an orthogonal synthetic approach as other reactive functional groups (BPin, Br, Cl, NO_2_, OTf) present on either coupling partner remained unscathed post reaction.

In 2012, Huo and co‐workers presented zirconium nucleophiles as an alternative to organozinc reagents.^[50]^ The heteroaromatic zirconium reagents were prepared in situ from oxidative addition of heteroaryl chlorides into Cp_2_ZrBu_2_. The sole example of a 2‐pyridyl zirconium coupling to an aryl bromide was obtained in moderate yield (56 %), thus further optimisation would be necessary to improve the applicability to the 2‐pyridyl problem.

**Scheme 8:**
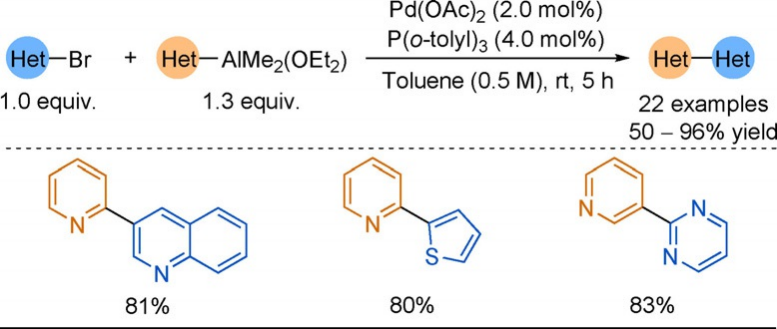
The 2‐pyridyl alane cross‐coupling with scope examples.

Arylalanes have gained popularity in C−C bond forming cross‐coupling reactions but with limited extension to the 2‐pyridyl problem. In 2014, Zhou reported the cross‐coupling of pyridyl or thienyl aluminium reagents with (hetero)aryl bromides and benzyl chlorides.^[51]^ The scope was limited to unsubstituted heteroaryl alanes, albeit in very good yields (Scheme 8). 2‐Pyridyl alanes coupled with consistently lower yields than the 3‐pyridyl substrates, reiterating the increased challenge associated with 2‐pyridyl nucleophiles. The reaction could be scaled up with in situ aluminium reagent preparation (5 mmol, 90 %, 3‐phenyl pyridine). 2,2′‐Bis‐azine could not be obtained, presumably due product inhibition of the catalyst.

Recently Sarandeses and co‐workers extended their pivotal triorganoindium‐palladium‐catalysed cross‐coupling methodology^[52]^ to include tri(pyridin‐2‐yl)indium nucleophiles, shown to couple efficiently to aryl, benzyl and alkenyl bromides (5 examples, 71–95 % yield).^[53]^ This was achieved by innovative development of stable, solid triorganoindium 4‐dimethylaminopyridine complexes.

### 2.5 2‐Pyridylsilanes (Si)

**Scheme 9:**
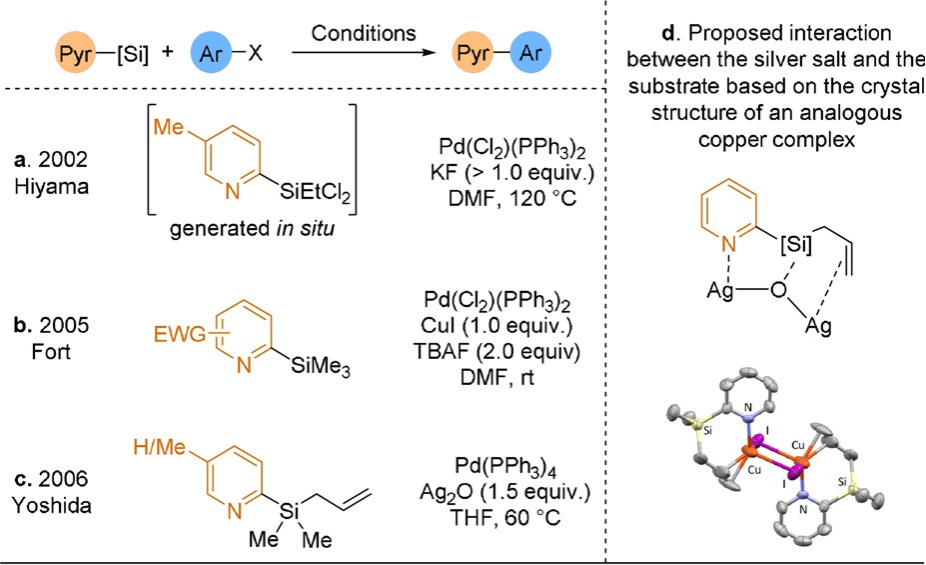
Developments of the Hiyama coupling to 2‐pyridyl substrates.

Organosilanes can also be used as nucleophilic coupling partners in desilylative coupling reactions with aryl halides. Hiyama was the first to report a cross‐coupling involving 2‐pyridylsilane nucleophiles using an unstable dichloroethylsilyl group (Scheme 9 a).^[54]^ In 2005, Fort and co‐workers reported the first stable and easy to handle 2‐pyridyltrimethylsilanes suitable for the Hiyama couping (Scheme 9 b).^[55]^ However, the scope was limited to pyridines bearing an electron‐withdrawing substituent to increase the polarisation of the C−Si bond, a problem that was solved by Whittaker and co‐workers using a silver additive.^[56]^ This was further improved by Yoshida and his group by replacing a methyl substituent with an allyl group on silicon (Scheme 9 c). A narrow scope of 2‐aryl pyridines (8 examples, 59–93 % yield) was obtained without need for any fluoride source.^[57]^ By analogy to the reported binding of CuI to 2‐(allyldimethylsilyl)pyridine,^[58]^ they proposed that the soft silver centre would bind to both pyridine and allyl moieties, while the hard oxygen atom would coordinate the silicon (Scheme 9 d). Under these conditions, other silyl groups such as homoallyl‐, vinyl‐, and *p*‐acetylbenzyl‐dimethylsilyl provided low to moderate reactivity, while 2‐(trimethylsilyl)pyridine remained unreactive, highlighting the poor polarisation of unactivated C−Si bonds.

**Scheme 10:**
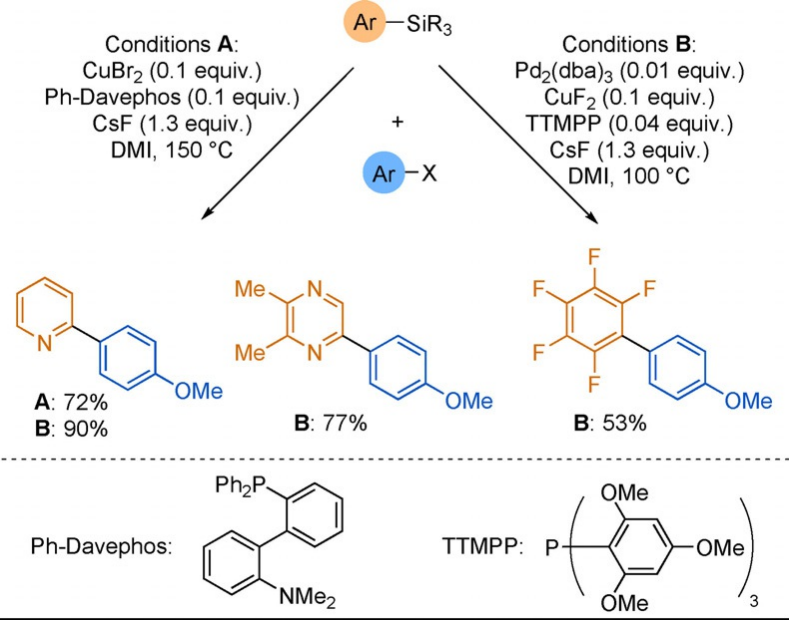
Novel catalytic systems for the coupling of aryltriethylsilanes with aryl bromides.

A range of other conditions were also developed for 2‐pyridyltrimethylsilane substrates, however, the substrate scope focused on the electrophile while the pyridine core remained poorly functionalised.^[59]^ The Hiyama group showed that 2‐pyridyltriethylsilanes could also be employed in a copper‐catalysed cross‐coupling reaction with aryl halides (Scheme 10, conditions A).^[60]^ The same group subsequently reported a dual Pd/Cu catalytic system which allowed the coupling of a range of silyl groups under milder conditions (Scheme 10, conditions B).^[61]^ The efficiency of these couplings was demonstrated with a large scope of heterocyclic substrates, as well as challenging polyfluorocarbocyclic nucleophiles, but the pyridine scope was once again limited.

Smith and co‐workers showed that isolation of the silane nucleophile could be circumvented by using elegant silicon‐based transfer agents.^[62]^ 2‐Lithiopyridine could therefore be used directly as the nucleophilic coupling partner, and the transfer agents could be recovered and reused without loss of reactivity or cross‐contamination. Together with the advancements in preparing heteroaryl silicon derivatives,^[63]^ the Hiyama coupling appears as a good alternative to traditional organometallic reagents. Unfortunately, the synthesis of 2‐pyridyl silanes still requires the use of organolithium reagents, and accessing functionalised 2‐pyridyl silane substrates remains a challenge.

## 3 Suzuki–Miyaura Cross‐Coupling

### 3.1 Problematic Suzuki–Miyaura Couplings

**Figure 2:**
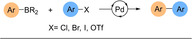
Schematic of the Suzuki–Miyaura cross‐coupling.

SMC reactions have emerged over recent decades as the favoured route for swift construction of C(sp^2^)−C(sp^2^) bonds, in both chemical industry and academia (Figure 2).[^9^, ^64^] The reason SMC has become the choice carbon−carbon bond forming methodology over more conventional organometallic cross‐coupling is in part due to milder reaction conditions, broad functional group tolerance and use of less toxic and more stable boron‐based nucleophiles.^[65]^ Boron‐nucleophiles have a less polarised carbon−metal bond than classical organometallic reagents. This generally allows better chemoselectivity and functional group tolerance.^[66]^ Another factor in the widespread use of SMC reactions is the continued research and innovation into development of new catalysts and boron‐reagents.[^64b^, ^67^] Additionally, these reactions have been developed alongside, and are compatible with, new emerging technologies, such as automation and microwave reactions.^[68]^

**Figure 3:**
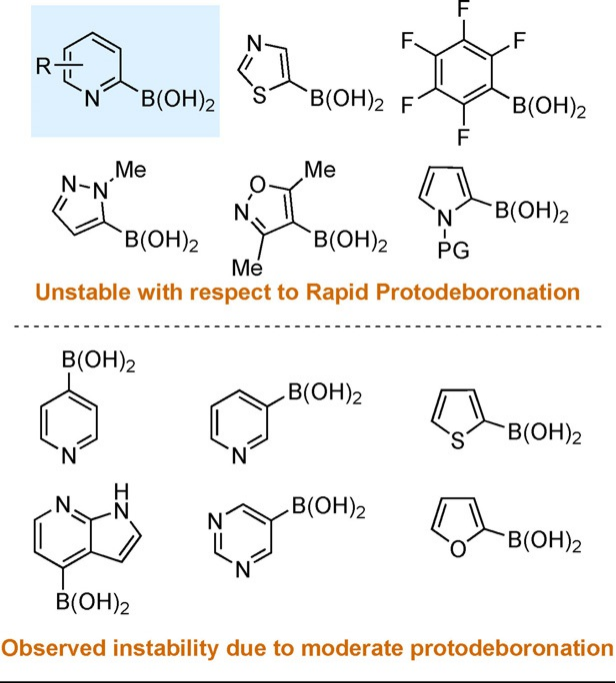
Boron‐reagents unstable towards protodeboronation (according to pH studies by Lloyd‐Jones and co‐workers).

Despite the wide‐spread application of boronic acids in SMC processes, some boronic acids have notoriously poor reaction success due to their instability, both under storage and SMC conditions. The most infamously unstable arylboronic acids with regards to protodeboronation are heteroaromatic (particularly 2‐heteroaryl) and polyfluorinated phenyls (Figure 3).^[19]^ These motifs are valued in industry and academia, making the difficulties in coupling these nucleophiles more frustrating. The cross‐coupling of 2‐pyridyl boron reagents is particularly challenging. Many reports using 2‐pyridyl boron nucleophiles show moderate to poor yields, limited scope of aryl or heteroaryl electrophiles, often require substrate specific optimisation, and employ boron reagents frequently prepared using organolithium chemistry.[^65^, ^69^] As a result, there is a sizeable body of research into establishing general SMC conditions for efficient coupling of these reagents and investigating novel, more stable 2‐pyridyl boron nucleophiles. Thus, the successful coupling of 2‐pyridyl boron reagents has become a benchmark for a robust SMC reaction.

In designing workable SMC solutions to the 2‐pyridyl problem, it is key to understand the challenges faced when coupling 2‐pyridyl boronates, particularly the innate propensity of these species to undergo protodeboronation.^[67a]^ Reports from the Kuivila group in 1961 gave initial mechanistic insights into the pathway of protodeboronation of arylboronic acids and the factors that influence the rate of decomposition.^[70]^ However, as these studies pre‐date the SMC reaction, the importance of pH in affecting the rate of decomposition was less explored. In 2014, Perrin and co‐workers published an investigation into base‐promoted protodeboronation of electron‐deficient (hetero)arylboronic acids.^[71]^ The report concluded that alkaline conditions rapidly accelerate the decomposition of 2,6‐dihalogen‐substitued arylboronic acids. Although no 2‐pyridyl boronic acids were studied, this highlighted the relationship between pH and the rate of protodeboronation.

**Scheme 11:**
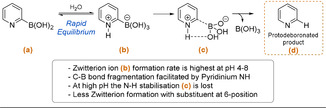
Proposed mechanism for the protodeboronation of 2‐pyridyl boronic acid.

Seminal work into understanding the instability of 2‐pyridyl boronic acid and boronates was reported by the group of Lloyd‐Jones.^[19]^ They investigated the pH‐dependent rate of protodeboronation for 18 unstable boronic acids, and proposed a general kinetic model.^[19a]^ 3‐ and 4‐pyridyl boronic acids were found to undergo slow protodeboronation under heating and basic conditions (*t*_1/2_>1 week, pH 12, 70 °C), whereas 2‐pyridyl and 5‐thiazolyl boronic acids undergo rapid protodeboronation under heating and neutral conditions (*t*_1/2_ 25–50 s, pH 7, 70 °C). The fast protodeboronation of 2‐pyridyl boronic acids was shown to not be accelerated by higher pH; instead, 2‐pyridyl boronic acid was more stable at high pH (pH >10) than under weakly acidic/basic conditions (pH 4–8). Lloyd‐Jones details how 2‐pyridyl boronic acids decompose via fragmentation of a zwitterionic intermediate, which is formed at a maximum rate between pH 4 and 8 (Scheme 11). This species is more readily formed from 2‐pyridyl boronic acids than the 3‐ or 4‐pyridyl analogues. This is partially attributed to the stronger ylidic character and closer charge placement in the zwitterion formed with 2‐pyridyl substrates. Zwitterionic fragmentation is strongly facilitated by the basic nitrogen adjacent to the boron, which stabilises the B(OH)_3_ leaving group during C−B bond cleavage. At higher pH this interaction is attenuated and protodeboronation is slower. The presence of this stabilising interaction explains in part why 2‐pyridyl species are especially prone to protodeboronation.

An electron‐withdrawing substituent at the 6‐position of 2‐pyridyl boronic acid results in protodeboronation occurring within a lower pH range than for the unsubstituted 2‐pyridine.^[19a]^ In addition, substituents at the 6‐position are proposed to block coordination of the pyridyl nitrogen to the Pd centre, thus preventing any reduction in catalytic activity from this interaction.^[72]^ This is worth noting, as multiple reports discussed in Section 3.3 give noticeably higher yields when the 2‐pyridyl nucleophile is 6‐substituted.

### 3.2 Advancement of SMC Conditions

#### 3.2.1 Developments in Catalytic Systems

Prior to in‐depth mechanistic understanding of protodeboronation, more active catalyst systems were employed as a strategy to circumvent boronate instability. SMC conditions were tailored to increase the rate of product formation, in order to outcompete protodeboronation. In the context of difficult SMC reactions the most notable families of ligands developed are bulky, electron‐rich monophosphines (e.g. SPhos, XPhos, PCy_3_)_,_ ^[73]^ and SPOs.^[74]^ Another strategy to improve the efficiency of a SMC catalytic system is to employ a precatalyst. There has been success in developing precatalysts that assist in enabling the use of milder conditions and/or shorter reaction times for the coupling of some challenging boronic acids such as polyfluorophenyls,^[75]^ 5‐membered heterocycles,[^75^, ^76^] and a handful of 6‐membered heteroaromatic boronates.[^76^, ^77^] However, use of these activated ligands and precatalyst systems alone does not provide a general solution to the 2‐pyridyl problem, although they are useful developments when used in conjunction with more stable boron‐derived reagents.[^75^, ^76^, ^77^]

#### 3.2.2 Copper Additives

**Scheme 12:**
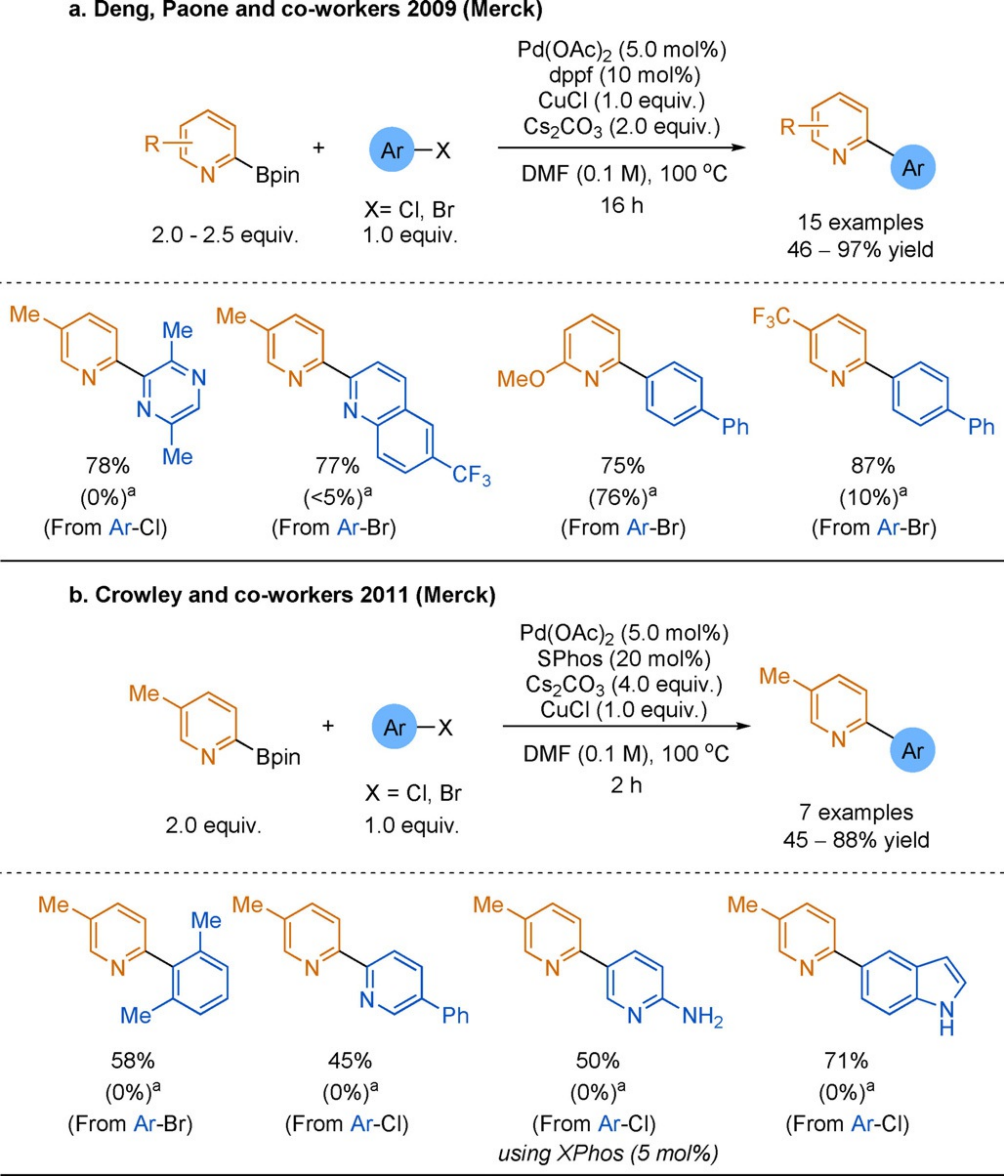
Copper‐assisted SMC of 2‐pyridyl Bpin. [a] No CuCl.

Lewis acidic metals, such as copper, silver and zinc, have historically been useful additives in conventional cross‐coupling reactions and have likewise had success in improving the yield of the SMC of particularly challenging nucleophiles.^[78]^ In 2009, Deng, Paone and co‐workers found that a stoichiometric copper additive was key in achieving high yields of 2‐arylpyridines when coupling various challenging 2‐heterocyclic pinacol boronates (Scheme 12 a).^[79]^ However, for 6‐substituted 2‐pyridyl boronates, the presence of copper was not necessary to obtain good yields. This is in line with the discussion of the relative stability of 6‐substituted 2‐pyridyl reagents in Section 3.1.

In 2011, Crowley and co‐workers expanded the scope of this copper‐assisted SMC by using S‐Phos or X‐Phos.^[80]^ This shortened the reaction times and allowed the use of less reactive aryl chlorides (Scheme 12 b). A drawback is that both methodologies require stoichiometric copper and a two‐fold excess of the boronate reagent to outcompete the competitive homocoupling of the 2‐pyridyl species. However, commercially available boronate esters and a cheap Cu^I^ source are used. Therefore, this approach does provide a straightforward solution to poor 2‐pyridyl boronate reactivity. The success of the copper additives reported here has been capitalised on in further reports using next‐generation boronate reagents, discussed in Section 3.3.

Concerning the role of copper, the authors postulated that the 2‐pyridyl boronate species first undergoes irreversible transmetalation to give a 2‐pyridyl cuprate in situ (Figure 4.1). This cuprate is proposed to undergo more efficient transmetalation with the active Pd species than the parent boronate, and circumvents the potential for protodeboronation. This is similar to one of the proposed roles of copper salts in Stille reactions.^[78d]^ However, in the aforementioned 2016 report from Lloyd‐Jones, the role of Lewis acid additives in preventing protodeboronation was extensively explored.^[19a]^ Through NMR studies, it was observed that copper binds reversibly to the pyridine (Figure 4.2). This Cu−N coordination reduces the proportion of the key zwitterion intermediate in the reaction mixture, which is responsible for protodeboronation. The authors concluded that it is the reversible complexation of copper to the pyridyl nitrogen which attenuates protodeboronation and improves reaction success. Compared to other Lewis acids (Zn, Ag, Sr), copper additives have the greatest impact, likely as copper is more azaphilic.

**Figure 4:**
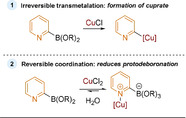
Proposed roles of copper in the SMC of 2‐pyridyl boronates. 1) Irreversible transmetalation. 2) Reversible coordination.

### 3.3 Alternative Boronate Species

**Figure 5:**
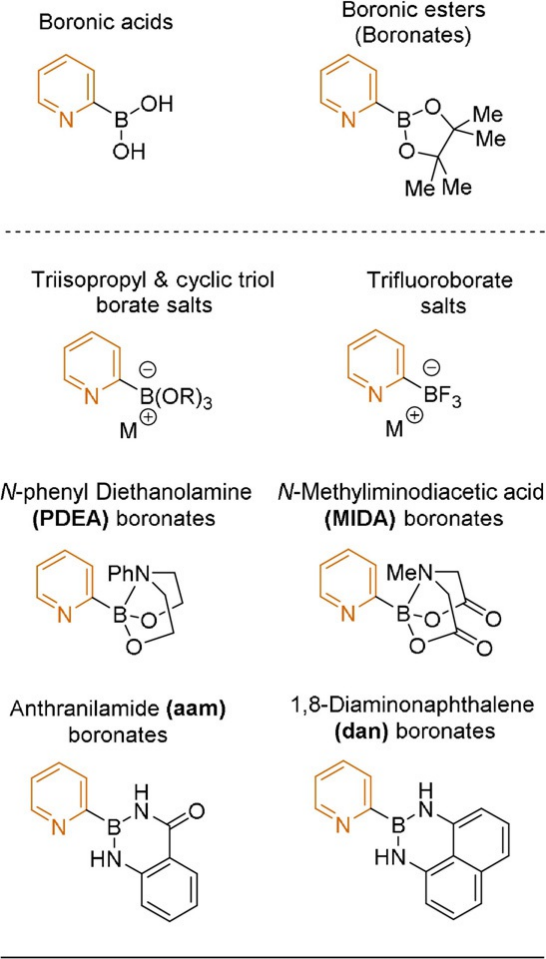
Boron‐derived 2‐pyridyl reagents.

As well as tailoring SMC conditions, another key focus area is the development of more stable boron‐derived reagents; ones that are resistant to, or undergo a slower rate of protodeboronation. A plethora of next‐generation organoboron nucleophiles have arisen over the last two decades (Figure 5).

These next‐generation boron‐based reagents operate either as slow‐release species or as more stable, direct coupling partners.[^66^, ^67^, ^81^] In both approaches, the Lewis acidity of the boron centre is reduced. The slow‐release strategy masks the boron centre, rendering it less reactive, and then, under the reaction conditions, the active boron species is released at a controlled rate.[^66^, ^67^, ^82^] This approach ensures that the ratio of catalyst to active, unmasked boron reagent is high and favours transmetalation over protodeboronation. The other, more recent approach is the development of stable boron nucleophiles that react directly in SMC reactions and do not hydrolyse to the boronic acid in situ.[^66^, ^81^]

There are multiple reviews discussing in depth the discovery, synthesis and development of various boron‐based nucleophiles.[^65^, ^67^, ^83^] However, in this Review, we focus on these species as applied to 2‐pyridyl couplings.

#### 3.3.1 Cyclic Triol and Triisopropyl Borate Salts

Aryl cyclic triolborates were first introduced for use in SMC by Miyaura in 2008.^[84]^ An advantage of triol salts over boronic acids is that they are bench‐stable complexes and are shown to be highly efficient in transmetalation. Miyaura established the use of these reagents in carbocyclic SMC couplings, boasting a large substrate scope. However, only one example of a 2‐pyridyl cyclic triolborate was featured, and the addition of CuI (20 mol %) was needed to achieve a high yield (90 %).

In 2010, Miyaura published a report focusing on heteroaromatic triolborates in SMC.^[85]^ Boronic acids that were challenging to couple under typical SMC conditions were shown to couple efficiently as triolborates using an aqueous base. Conditions were optimised for the SMC of 2‐pyridyl, 3‐pyridyl or 2‐thiophenyl triolborates, and a small scope was established. More general conditions followed in 2011, however, the scope of 2‐pyridyl substrates was again limited.^[86]^ In 2012, Cefalo and co‐workers reported a system for coupling lithium triisopropyl‐ and triol‐2‐pyridylborate salts, involving dual addition of catalytic CuCl and stoichiometric ZnCl_2_ to the Pd‐mediated reaction.^[78c]^ However, the scope was small and low yielding. Notably, Cu^I^ additives again proved essential for improving the reaction in all these reports.

**Scheme 13:**
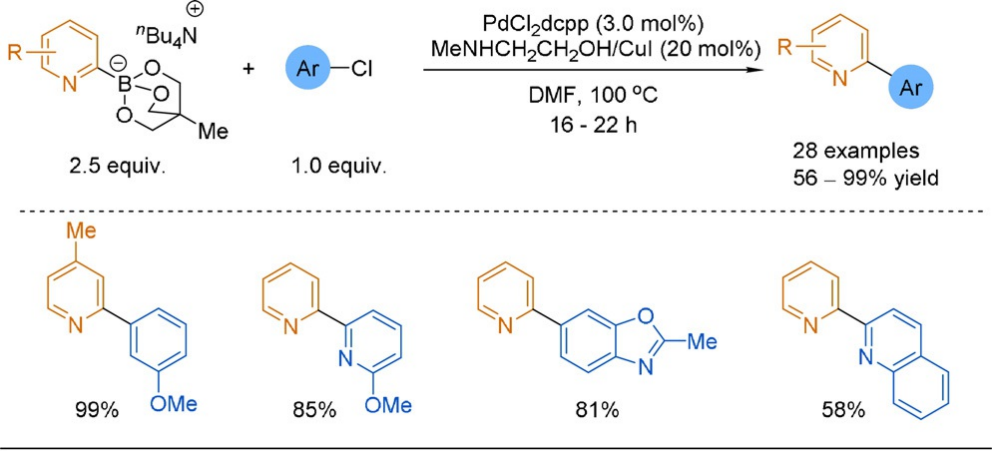
Use of TBA 2‐pyridyltriolborate salts in SMC. dcpp=1,3‐bis(dicyclohexylphosphino)propane.

Traditionally the counterion for cyclic triolborates is potassium or lithium.[^84^, ^85^, ^86^] This limits solubility of these reagents in organic medium. In 2013, Yamamoto and co‐workers introduced tetrabutylammonium (TBA) 2‐pyridyltriolborate salts for use in SMC.^[87]^ The rate of the transmetalation step was observed to be faster with the TBA salt compared to other alkali metal counterions (Bu_4_N^+^>Cs^+^>K^+^>Na^+^>Li^+^). This higher reactivity is what the authors reason enables the use of less activated aryl chlorides as the electrophiles. Indeed, a large scope of 2‐(hetero)arylpyridines has been shown, with good to excellent yields (Scheme 13). Notably, no base was used. Instead, an amino ligand was employed in conjunction with a copper additive. This methodology is a useful addition to the tools for the 2‐pyridyl problem, however, there are some drawbacks. Namely, there is poor atom economy in using TBA salts, which are used in excess and are also non‐commercially available fragments.

As previously discussed (Section 3.2.1.), copper helps to attenuate the major decomposition pathway of 2‐pyridyl boronates.[^19a^, ^79^] Hence, the use of a copper additive in all these reports calls into question if the more efficient coupling of 2‐pyridyl borates can be attributed to the cyclic triolborate or the copper.

**Scheme 14:**
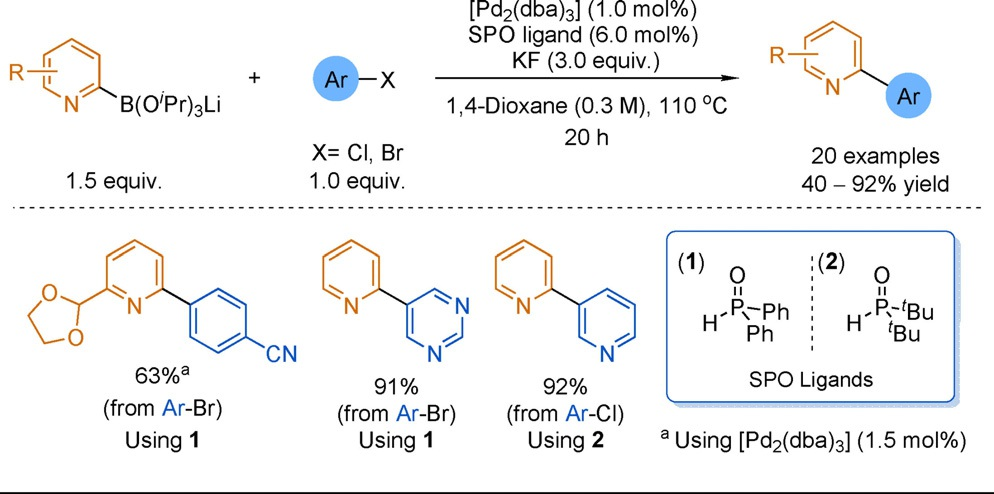
Selected scope from the SMC of lithium 2‐pyridyl−B(O^*i*^Pr)_3_ reagents.

In 2008, Billingsley and Buchwald presented a general method for the SMC of 2‐pyridyl triisopropyl borates (B(O^*i*^Pr)_3_) using SPO ligands.^[74b]^ In this report, various 2‐pyridyl boron reagents were used and quantitative conversion was seen only when employing lithium 2‐pyridyl−B(O^*i*^Pr)_3_ reagents (Scheme 14). In order for aryl chlorides to be coupled, the bulkier and more electron‐rich ligand **2** had to be used. Unlike the cyclic triolborate work, this system does not require a Cu additive. Potukuchi and Ackermann similarly reported the reaction of various substituted 2‐pyridyl−B(O^*i*^Pr)_3_Li reagents with aryl bromides using SPO ligands, also without the aid of copper (19 examples, 30–87 %).^[88]^

Overall, methodologies utilising 2‐pyridyl triisopropyl‐ and triol‐borates require specific tuning to be of use as solutions to the 2‐pyridyl problem, demanding large organic counterions, specific phosphine oxide ligands or metal additives. Moreover, the primary routes to access these species proceed via the unstable parent boronic acid, or are functional group restricted as they involve lithiation.[^74b^, ^85^, ^87^, ^88^]

#### 3.3.2 N‐Phenyldiethanolamine Boronates (PDEA)

In 2004, Hodgson and Salingue developed a novel amino‐stabilised boronate for 2‐pyridyl couplings using a N‐phenyldiethanolamine (PDEA) group.^[89]^ PDEA boronates are stabilised by the intramolecular dative bond between the nitrogen and boron atoms. As a result these reagents are stable to prolonged storage. The authors showed that 2‐pyridyl−B(PDEA) could be synthesised in a scalable, one‐pot procedure from the 2‐bromopyridine via the triisopropyl borate in good yields. In this report, the first SMC system specifically optimised for the coupling of 2‐pyridyl−B(PDEA) with aryl bromides and iodides was described.^[89]^ A small scope of nine 2‐arylpyridines was obtained in varied yields (10–89 %); no biheteroaryls were prepared. Addition of copper was essential, again challenging how much of the improved reaction efficacy is due to the stabilised boronate versus the Cu^I^ salt.

**Scheme 15:**
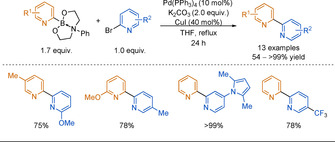
Selected examples of the coupling of PDEA boronates.

In 2007, Steven and co‐workers accessed a moderate scope of 2‐aryl‐pyridines (10 examples, 47–84 % yield) using B(PDEA) under similar conditions to those reported by Hodgson.^[69a]^ Although only the unsubstituted 2‐pyridine boronate was used, the improved conditions allowed the coupling of various less reactive heteroaromatic electrophiles. In 2010 Lützen and Gütz published a more extensive investigation,^[90]^ and a range of 2,2′‐bipyridines were synthesised in comparable, or even better yields than the same products accessed using Negishi or Stille cross‐coupling reactions (Scheme 15).

The use of 2‐pyridyl PDEA boronates has been successfully adapted to solid support chemistry.^[91]^ As before, a copper additive was essential.

#### 3.3.3 N‐Methyliminodiacetic Acid (MIDA) Boronates

In 2007, Burke and Gillis introduced the use of a boronic acid protected by the trivalent N‐methyliminodiacetic acid (MIDA) ligand for use in iterative SMC.^[92]^ Initially, B(MIDA) reagents were used as masking groups, being converted into the parent boronic acid on treatment with aqueous base.

**Scheme 16:**
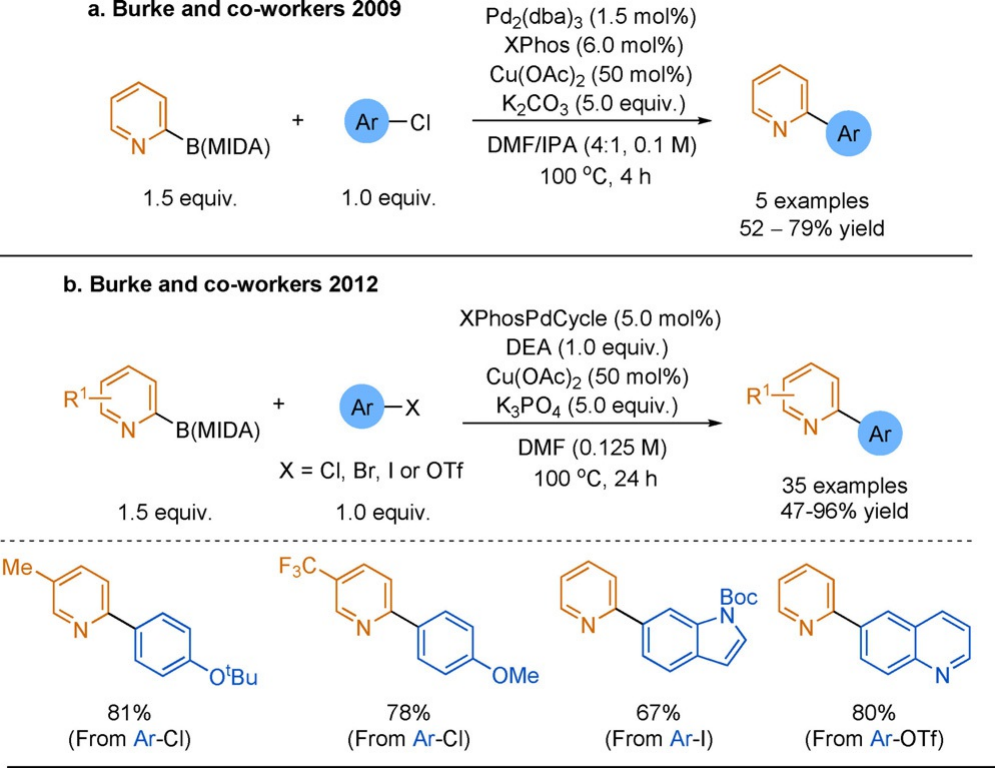
Use of 2‐pyridyl−B(MIDA) in SMC reactions.

In 2009, Burke and co‐workers drastically expanded the utility of B(MIDA) reagents by using them directly in SMC.^[82b]^ As previously discussed, there is kinetic competition between in situ protodeboronation of unstable boronic acids and their cross‐coupling. As a solution Burke and co‐workers devised a “slow‐release” strategy. This denotes the controlled rate of formation of unstable boronic acids from (bench‐stable) B(MIDA) in situ.^[67a]^ The stabilising B−N coordination in B(MIDA), similarly seen in PDEA boronates,^[89]^ is key for allowing the slow release to be achieved. MIDA boronates hydrolyse quickly when treated with strong base such as NaOH (<10 min at 23 °C).^[82b]^ Utilising a weaker base, tailoring the solvent system, and controlling the reaction temperature enabled the hydrolysis of B(MIDA) to be moderated. This strategy was applied to a range of challenging boron nucleophiles, including the unsubstituted 2‐pyridyl−B(MIDA) (Scheme 16 a). The product yields were dramatically higher using B(MIDA) compared to the boronic acid directly. In support of the slow‐release hypothesis, increasing the rate of B(MIDA) release through use of a strong base (aqueous NaOH) gave similar yields to direct use of the boronic acid. Notably, a copper additive was employed.

In 2012 Burke and co‐workers reported a SMC system specifically tailored for 2‐pyridyl MIDA boronates, offering this as “the first general solution to the 2‐pyridine problem”.^[93]^ The combination of a XPhos palladacycle G1 catalyst and copper diethanolamine (DEA) as an additive were found to be optimum. The authors demonstrated the generality of these SMC conditions by obtaining a large scope of biaryls in good yields, using both activated and deactivated (hetero)aryl halides and triflates (Scheme 16 b). Similar to the synthesis of previously described boron reagents, a scalable method for preparing 2‐pyridyl−B(MIDA) reagents was also detailed: accessing triisopropylborates via lithiation, followed by ligand exchange.

First reported by Lipshutz and co‐workers in 2013, the use of a designer surfactant in the SMC of aryl−B(MIDA) enabled high product yields to be obtained under aqueous nanomicellar conditions at room temperature.^[94]^ Micelle catalysis allows the coupling to run under mild conditions, theoretically in small apolar aggregates, therein avoiding fast protodeboronation. Although not the focus of the paper, preliminary studies showed 6‐methoxy‐2‐pyridyl MIDA boronates were amenable to micellar catalysis.

**Figure 6:**
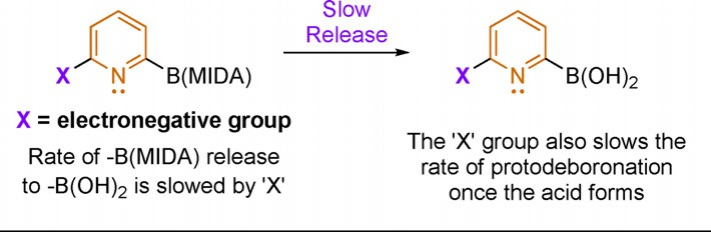
The proposed “attenuation” strategy.

In 2017, Lipshutz reported the application of micellar catalysis directly to 6‐substituted 2‐pyridyl−B(MIDA).^[72a]^ One of the proposed roles of Cu additives in SMC is that Cu coordinates the pyridyl nitrogen and prevents unproductive Pd−N coordination.^[19a]^ The authors propose that, instead, Pd−N coordination could be sterically blocked by a substituent at the 6‐position (Figure 6). To maintain the versatility of this method, the authors demonstrated that the substituent placed in the 6‐position of the 2‐pyridyl boronate could be easily removed or further transformed after cross‐coupling.

**Scheme 17:**
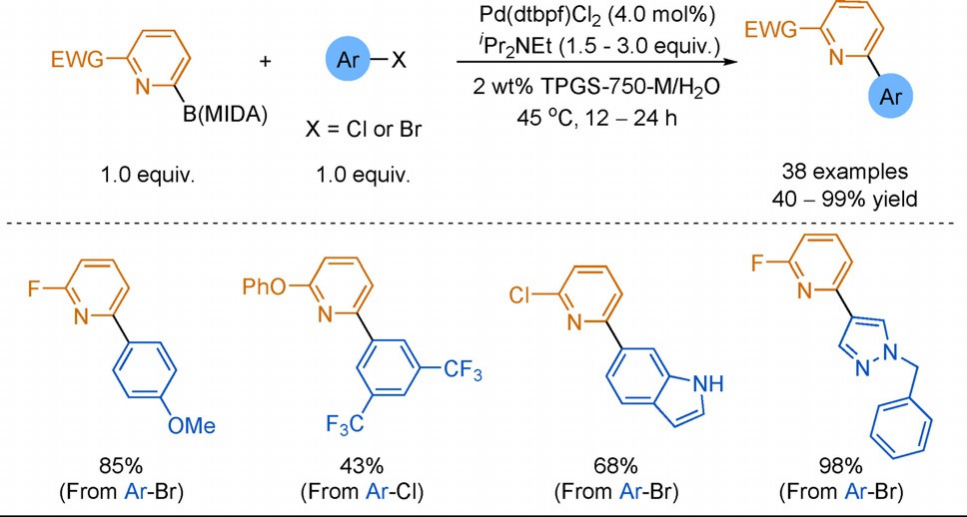
Micelle‐catalysed coupling of 6‐substituted pyridyl−B(MIDA).

In addition to attenuating Pd−N(pyridine) coordination, computational data shows that an electronegative group in the 6‐position promotes cross‐couplings by reducing the rate of protodeboronation of the 2‐pyridylboronic acids formed in situ.^[72a]^ The scope of the reaction is broad, with a diverse scope of 2‐hetero(aryl)pyridines achieved (Scheme 17). Furthermore, no homocoupling of the B(MIDA) was observed. The combination of micelle catalysis and the attenuation strategy was further developed by Novartis chemists in 2018, who reported a modest scope of biheteroaryls from the SMC of 6‐chloro‐2‐pyridyl Bpin.^[95]^

Ligand‐free conditions have also been developed for the use of 2‐pyridyl−B(MIDA),^[96]^ however a substituent in the 6‐position of the pyridine is required.^[97]^

#### 3.3.4 Organotrifluoroborates

Potassium organotrifluoroborates (R−BF_3_K) are another class of popular nucleophilic reagents for SMC reactions. These reagents are air and moisture stable, and can be prepared easily from organoboron reagents and cheap potassium hydrogen fluoride (KHF_2_).[^83^, ^98^] The use of Ar−BF_3_K reagents in challenging SMC reactions was considerably advanced by the Molander group in the early 2000s.^[99]^ In 2003, they demonstrated that many (hetero)aryl–aryl scaffolds could be constructed through coupling of (hetero)aryl−BF_3_K with aryl halides under ligandless conditions. However, the group explicitly showed that the coupling of 2‐pyridyltrifluoroborate reagents was unsuccessful.

**Scheme 18:**
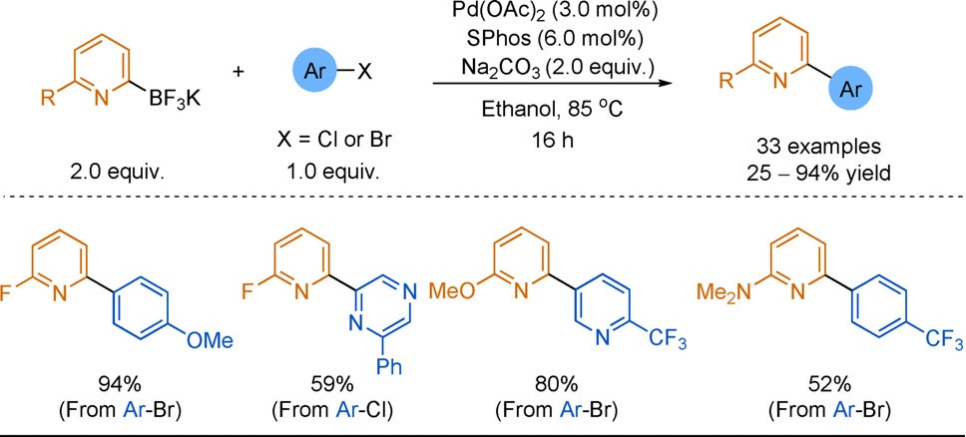
The coupling of 2‐pyridyl−BF_3_K reagents.

Only in 2012 did Wu and co‐workers report optimised conditions for the SMC of 2‐pyridyl−BF_3_K reagents.^[100]^ Similarly to other research into the 2‐pyridyl problem using the SMC reaction, an electron‐rich and bulky monophosphine ligand was used. The authors report a broad range of 2‐(hetero)arylpyridines synthesised in moderate to good yields (Scheme 18). Although the scope of the electrophilic partner is broad, only 6‐substituted 2‐pyridyl‐trifluoroborates were coupled. Additionally, there are innate disadvantages with these reagents; as salts they are often difficult to purify, to progress through multi‐step synthesis, and have limited solubility in organic media.

**Scheme 19:**
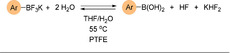
The slow‐release mechanism of aryl−BF_3_K reagents.

Studies of the cross‐coupling of carbocyclic aryl−BF_3_K with aryl bromides show that the reaction proceeds via hydrolysis of the borate in situ (Scheme 19).^[101]^ The boronic acid is the species that actively joins the catalytic cycle.^[101a]^ Optimisation of the system is often necessary for each class of substrate, which is likely due to the need to balance the rate of hydrolysis with the rate of catalyst turnover, as seen with MIDA boronates.^[101b]^ Thus, the efficacy of aryl−BF_3_K reagents in achieving coupling where the analogous boronic acid is unstable is credited to the slow‐release strategy.^[67a]^

In principle, the slow‐release approach appears a promising solution to the 2‐pyridyl problem. However, outside of the previously discussed reports, examples of 2‐pyridyl−B(MIDA) and −BF_3_K reagents being used to prepare highly functionalised 2‐arylpyridines are not common. The use of these 2‐pyridyl boronic acid surrogates in synthesising bioactive structures has seen varying degrees of success.^[102]^

#### 3.3.5 Anthranilamide (aam) Boronates

Anthranilamide (aam)‐substituted arylboranes were first introduced by Suginome and co‐workers in 2011.^[103]^ These aam units were originally developed as boron protecting groups, enabling a boron centre to be carried through multistep synthesis before being selectively deprotected to the boronic acid in the presence of acid. Aryl−B(aam) are reasonably moisture and air stable, although they are more prone to hydrolysis than both the corresponding B(dan) and B(MIDA) reagents.^[103]^

**Scheme 20:**
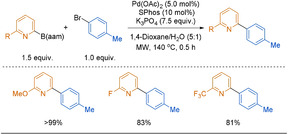
Direct SMC reaction of 2‐pyridyl−B(aam) reagents.

In 2019, aryl−B(aam) was first used directly in a microwave‐assisted SMC coupling by Yoshida and co‐workers.^[104]^ The authors propose that aryl−B(aam) acts as a slow‐release reagent, releasing the active boronic acid in situ, thus removing the need for stepwise acidic deprotection. Indeed, the reaction is most efficient in an aqueous medium, whereas no reaction is observed under anhydrous conditions, thus supporting the slow‐release postulate. A small scope of 2‐arylpyridines were reported in high yields, albeit using elevated temperatures (Scheme 20). Notably, only 6‐substituted‐2‐pyridyl−B(aam) reagents were used. Other heteroaryl−B(aam) reagents (2‐thienyl and 2‐furyl) were also shown to couple smoothly under these conditions. Although a weak base is employed, it is noteworthy that a large excess is needed. In contrast to previous slow‐release boronates, addition of Cu(OAc)_2_ did not significantly promote the reaction.

To illustrate the stability of 2‐pyridyl−B(aam) reagents, the authors noted that no decomposition was found in a batch of 6‐methoxy‐2‐pyridyl−B(aam) stored at ambient temperature, 1.4 years after its synthesis. Although still in a nascent state of application to the 2‐pyridyl problem, B(aam) reagents hold promise for further application.

#### 3.3.6 1,8‐Diaminonaphthalene (dan) Boronates

Another amino‐stabilised boron reagent that has recently gained traction is the 1,8‐diaminonaphthalene (dan)‐protected arylboronic acid. Alike B(aam), dan boronates were originally introduced by Suginome and co‐workers for use in iterative SMC reactions.^[105]^ Originally developed as a boronate masking group, the dan group was intended to make the reactive boron centre inert under SMC conditions. The B(dan) reagent would then be subjected to a separate acid deprotection step to reveal the active boron species.^[105]^ Likely due to the donation of the diamino group nitrogen lone pair into the vacant p‐orbital on boron, B(dan) reagents are considerably more stable than the corresponding aryl−B(MIDA) towards hydrolysis.^[103]^

Although introduced in 2007, aryl−B(dan) was not used directly in SMC reactions until 2020.[^66^, ^81^] In these recent reports, aryl−B(dan) is highlighted as stable with regards to protodeboronation. Unlike other slow‐release boronates, B(dan) reacts directly in the SMC reaction and does not hydrolyse to the boronic acid in situ. Indeed, ^11^B NMR studies confirm that the intact B(dan) species is transmetalation active.^[66]^

**Figure 7:**
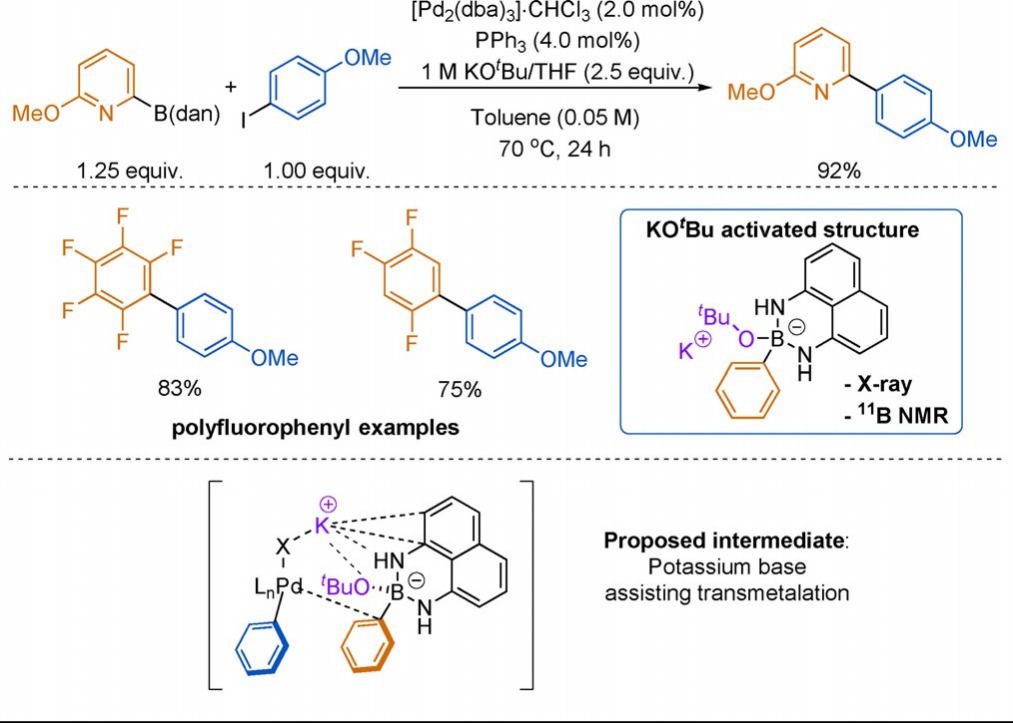
Coupling of Aryl−B(dan) and the role of KO^*t*^Bu.

Alongside various carbocyclic polyfluorophenyl substrates, Saito and co‐workers reported a singular example of 2‐pyridyl−B(dan) coupling under SMC conditions (Figure 7).^[81]^ Notably, 2‐pyridyl−B(dan) and polyfluorophenyl−B(dan) reagents were demonstrated to be stable and easily purified by flash column chromatography. The use of KO^*t*^Bu was key to reaction success, as it enables the formation of the butoxide–borate complex, which is proposed to efficiently undergo transmetalation with palladium, as well as capturing the halide leaving group. Notably, anhydrous conditions were favoured, further supporting that B(dan) is not hydrolysed to the boronic acid in situ.

**Scheme 21:**
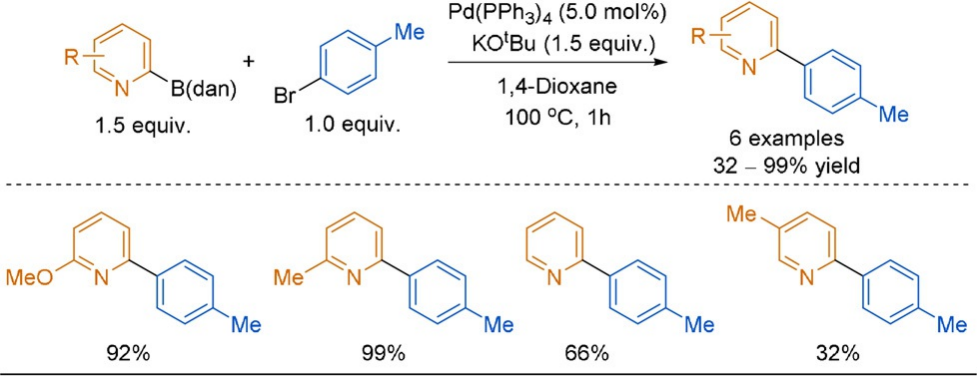
Direct coupling of 2‐pyridyl−B(dan).

Published concurrently with the above work, Tsuchimoto and Yoshida also showed the direct use of aryl−B(dan) in cross‐coupling reactions.^[66]^ The reaction conditions reported are similar and also require KO^*t*^Bu as the base for reaction success. The authors similarly cite the necessary generation of the shown active borate species. The main difference is the use of a more polar solvent and increased temperature, enabling a much shorter reaction time. Unlike the report by Saito and co‐workers, the scope featured multiple 2‐pyridyl−B(dan) substrates with varying substitution patterns, although high yields were only obtained for 6‐substituted‐2‐pyridyl−B(dan) reagents (Scheme 21). Aryl−B(dan) reagents can be synthesised through similar methods to the other masked boronates described previously.[^66^, ^81^, ^106^]

These reports present B(dan) as a promising complementary solution to the 2‐pyridyl problem, and an alternative to slow‐release boron reagents. However, the direct use of 2‐pyridyl−B(dan) is still in its infancy, and exploration into more challenging 2‐pyridyl couplings, particularly hetero−hetero couplings, is yet to be seen.

## 4 Alternative Approaches

### 4.1 Decarboxylative Cross‐Couplings

Decarboxylation has been studied since the early 20th century,^[107]^ with the very first decarboxylative cross‐coupling documented by Nilsson in 1966.^[108]^ The benefits of using carboxylate nucleophiles in coupling reactions are that they are readily available as well as cheap, generally non‐toxic, stable at ambient temperature and can be considered a green alternative to the corresponding sensitive and costly organometallic reagents. These advantages have attracted the scientific community, and the last few decades have seen many developments in decarboxylative cross‐coupling chemistry.^[109]^

**Scheme 22:**
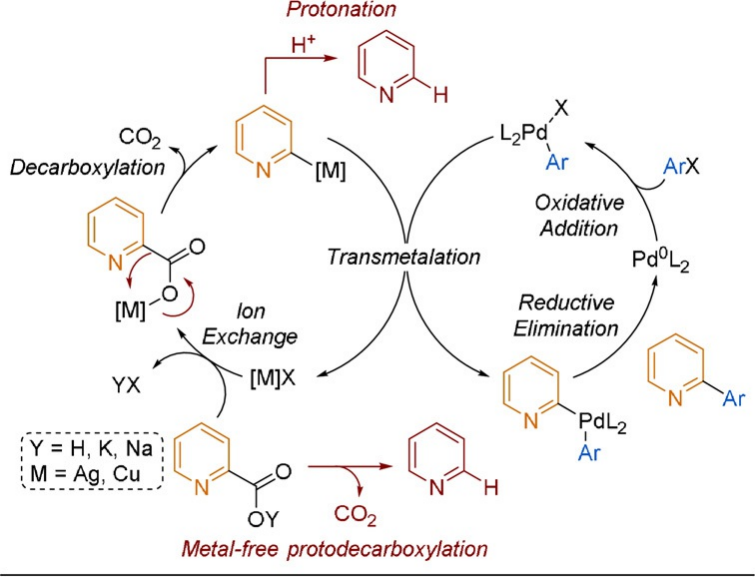
Representative picolinic acid decarboxylative cross‐coupling catalytic cycle with competing pathways.

Extending decarboxylative methodology to electron‐deficient heteroaryl nucleophiles has proved challenging, especially for pyridyl substrates. Standard conditions can be applied to 3‐pyridyl carboxylic acids, albeit with low yields,^[110]^ however, 4‐pyridyl carboxylic acids require tailored catalytic systems.^[111]^ These difficulties pale in comparison to 2‐pyridyl carboxylic acids, which have a propensity to protodecarboxylate.^[112]^ For an efficient decarboxylative cross‐coupling process, protodecarboxylation needs to be avoided and the high activation barrier of the metal‐mediated decarboxylation lowered (Scheme 22). In this section, efforts towards achieving this will be discussed.

**Scheme 23:**
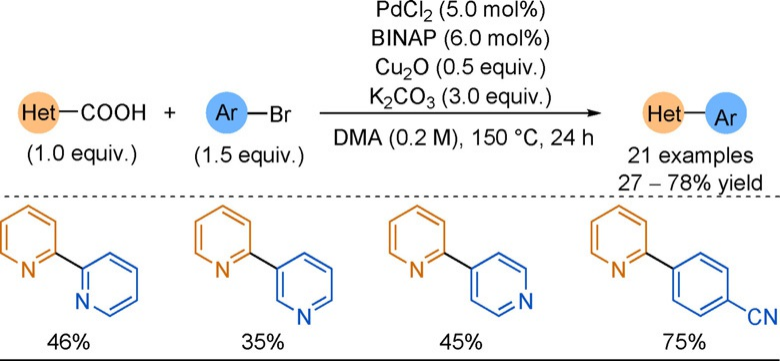
Decarboxylative cross‐coupling scope. BINAP=2,2′‐bis(diphenylphosphino)‐1,1′‐binaphthalene.

The first example of palladium‐catalysed decarboxylative cross‐couplings between 2‐picolinic acids and (hetero)aryl bromides was presented by Wu and co‐workers in 2013 (Scheme 23).^[113]^ In this seminal work, the authors reasoned that a bidentate ligand, with a rigid bite angle, helped suppress homocoupled byproduct formation, however low yields were still attributed to this issue plus formation of protodecarboxylated pyridine. Both silver and copper salts are commonly used additives in decarboxylative couplings; in this work copper(I) salts proved more efficient than silver salts. The scope featured only unsubstituted picolinic acid as the carboxylate coupling partner, sterically hindered electrophiles and those with carbonyls gave reduced yields, and aryl bromides were required for effective coupling; iodides led to homocoupling, and chlorides were inactive under the reaction conditions.

Stoltz and co‐workers presented a similar dual Pd/Cu‐catalysed decarboxylative coupling using potassium picolinate as the nucleophile.^[114]^ In this work, higher reaction temperatures (190 °C) were required to promote the challenging metal‐mediated decarboxylation, however, these harsh conditions also led to significant byproduct formation. Although in these two formative works the biheteroaryl products were not obtained in high yields, 2‐picolinic acids were shown to be viable 2‐pyridyl nucleophiles.

**Scheme 24:**
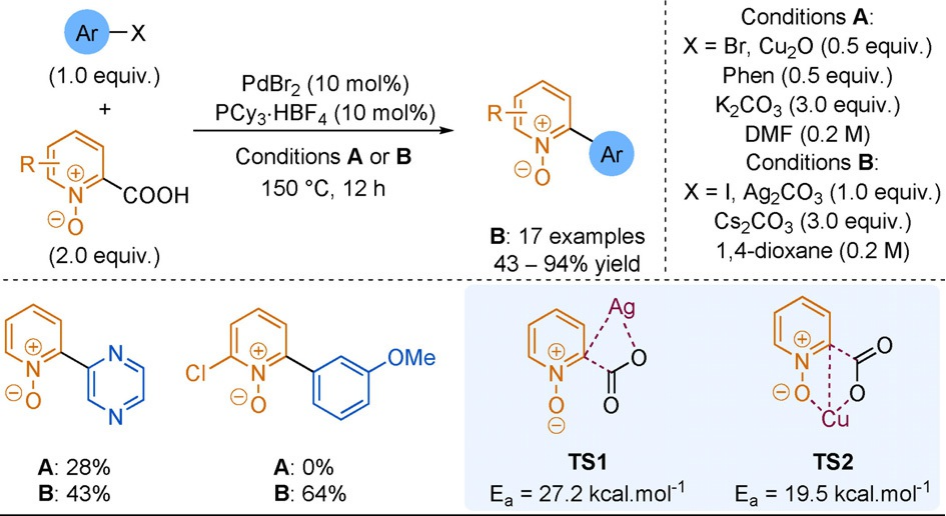
N‐oxide decarboxylative cross‐coupling examples showing that [Ag] outperformed [Cu]. Transition states investigated by DFT calculations for the decarboxylative‐metalation step.

An alternative approach was to use pyridine N‐oxides, which are more reactive than pyridines (see section 5), with carboxylic acid functionality in the 2‐position. The first decarboxylative cross‐coupling of picolinic acid N‐oxides with aryl halides via bimetallic catalysis was reported by Hoarau and co‐workers in 2014.^[115]^ Couplings to heteroaryl halides gave products in moderate to good yields, with the scope not limited to unsubstituted pyridine N‐oxides (Scheme 24). Although a high temperature (150 °C) was still required, the authors demonstrated a broader range of substrates in higher yields than those shown in previous reports. Alike the work by the groups of Wu and Stoltz, protodecarboxylation was found to be a dominating side reaction in the Cu‐mediated process. Further mechanistic insight was gained computationally; increased interactions to the small copper metal centre significantly lowered the decarboxylation activation energy (*E*_a_) compared to silver (Scheme 24). This lowered *E*_a_ led to accumulation of the easily protonated decarboxylated Cu‐intermediate (Scheme 22). Due to the tendency towards protodecarboxylation for the Cu system, higher yields were unsurprisingly observed with silver (Scheme 24). This process is reminiscent of the fast protodeboronation of 2‐pyridyl boronic acids described in section 3.2.

While picolinic acid N‐oxide is a cheap, commercially available substrate, the additional synthetic steps required to prepare more complex N‐oxides that are not widely available are a drawback. Furthermore, an extra step is needed to deoxygenate the products after coupling, which makes the process more inefficient and less atom economical.

**Scheme 25:**
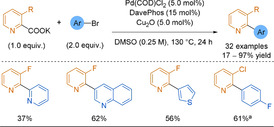
Selected scope examples. [a] Ag_2_CO_3_ (5 mol %) used instead of Cu_2_O.

In 2017, Gooßen focused on decarboxylative cross‐coupling of 3‐fluoro‐2‐picolinic acid potassium salts; subsequent nucleophilic aromatic substitution could lead to other useful pharmacophores.^[116]^ Unlike earlier work described in this Review, the metal additive could be employed in substoichiometric amounts. However, homocoupling and picolinic acid protodecarboxylation were once again key side reactions. Interestingly, during reaction optimisation several phosphines underwent aryl group scrambling with the reagents after P−C bond cleavage to give 2‐arylpyridine products. This exact process was later exploited by McNally and will be discussed in Section 4.3. In general, the reaction was tolerant to a broad range of functionalised electrophiles, but coupling more inactive aryl chlorides instead of bromides substantially reduced yields. Alike other decarboxylation work, acyl groups were not well tolerated (31–52 %) owing to interfering Cu coordination. The decarboxylation operated with heterocyclic bromides in moderate to good yields (Scheme 25), and the scope was not limited to 3‐fluoropicolinic acids, although tailored reaction conditions were required for some substrates. Unsubstituted picolinic acids required extreme temperatures of 190 °C, comparable to the work of Stoltz, and resulted in poor yields (28–40 %). A substituent next to the carboxylate is known to facilitate the decarboxylation of benzoates,^[117]^ but this finding also shows it is of importance for picolinates. Any other substitution pattern on the picolinate resulted in no product formation.

Continued improvement in the environmental impact of these 2‐pyridyl decarboxylative processes is necessary, such as lowering the temperature of metal‐catalysed reactions and avoiding the use of undesirable polar aprotic solvents and bases (such as DMF, pyridine).^[118]^

### 4.2 Desulfinative Cross‐Couplings

Desulfinative cross‐couplings have been explored over the last few decades, primarily focusing on aryl sulfinates.^[119]^ Conceptually, desulfinative coupling processes are similar to decarboxylation. Sulfinate salts can be obtained through various methods, including oxidation of thiols, reduction of sulfonyl chlorides and insertion of SO_2_ into a metalated species (Mg or Li) by use of an organic SO_2_ surrogate (e.g. DABSO).^[120]^ Furthermore, similarly to carboxylates, many sulfinate salts are inexpensive, commercially available and exhibit lower toxicity profiles^[121]^ than traditional organometallic reagents.

Electron‐poor heterocyclic sulfinate nucleophiles were underexplored in desulfinative cross‐couplings until the Willis group reported pyridine‐2‐sulfinates as efficient alternatives to 2‐pyridyl boronic derivatives in SMC (Scheme 28).^[122]^ Couplings to less active and cheaper aryl chlorides were equally as efficient as those to the corresponding bromides, with the process producing high yields of pharmaceutically relevant heteroaryl pyridines that would have been challenging to synthesise by classical methods (38 examples, including coupled pyrimidines, quinolines, pyrazines). In a second report, the temperature of the reaction could be lowered to 120 °C owing to the use of a bulkier and more electron rich ligand, improving the functional group tolerance of the process.^[123]^ This reduced reaction temperature demonstrates an advantage over decarboxylation.

**Scheme 26:**
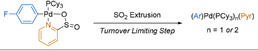
Extrusion of SO_2_ from 2‐pyridyl sulfinate palladium complexes.

Studies into the mechanism suggested that the potassium carbonate has two roles:[^72b^, ^124^] The potassium undergoes a cation metathesis with the sodium sulfinate salt which facilitates the transmetalation step, while the carbonate traps the SO_2_ byproduct and permits catalyst turnover. Alike the issues associated with picolinic acid decarboxylation, the nitrogen of the pyridine sulfinate strongly chelates to the Pd centre. Loss of SO_2_ from this complex is turnover limiting (Scheme 26). Hence, high reaction temperatures, particularly for 2‐pyridyl substrates, are required to overcome this strong κ^2^_N,O_‐chelation.

**Scheme 27:**
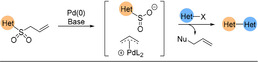
Simplified 2‐pyridyl allylsulfone desulfinative cross‐coupling mechanism.

While pyridine‐2‐sulfinates are an excellent tool in forming medicinally relevant cross‐coupled biaryl products, they are not without issue. Being salts, they display purification and solubility issues in organic media, which can in turn limit their utility. In 2018, the Willis group described allylsulfones acting as latent sulfinate reagents.^[125]^ The Pd catalyst has a dual function; first, the sulfinate “unmasks” in situ through deallylation and then, the previously described desulfinative cross‐coupling process follows (Scheme 27).

**Scheme 28:**
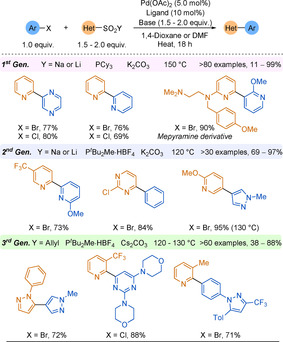
Summary of Willis group desulfinative cross‐coupling reactions.

The allylsulfone demonstrated orthogonal reactivity to SMC and could withstand functional‐group interconversions on the pyridine core highlighting the stability of this functionality. As well as pyridyl nucleophiles, the scope featured couplings of challenging 5‐membered rings (pyrazoles, imidazole, isoxazole) and heterocyclic cores of medicinal agents (e.g. COX‐2 inhibitors). The Willis group desulfinative cross‐couplings to form biheteroaryls are summarised in Scheme 28.

### 4.3 Main Group Ligand Couplings

In transition‐metal catalysis, ligand scrambling and aryl transfer to phosphine ligands are often seen as side‐reactions to be avoided rather than posing synthetic utility.^[126]^ However, the formation of biheteroaryl products through phosphorus centres (phosphorus ligand couplings) has been reported as early as the 1940s. These early methods are mostly restricted to homocouplings and do not feature a set of conditions suitable to a wide range of substrates.^[127]^ Over recent years, this chemistry has had a resurgence.

**Scheme 29:**
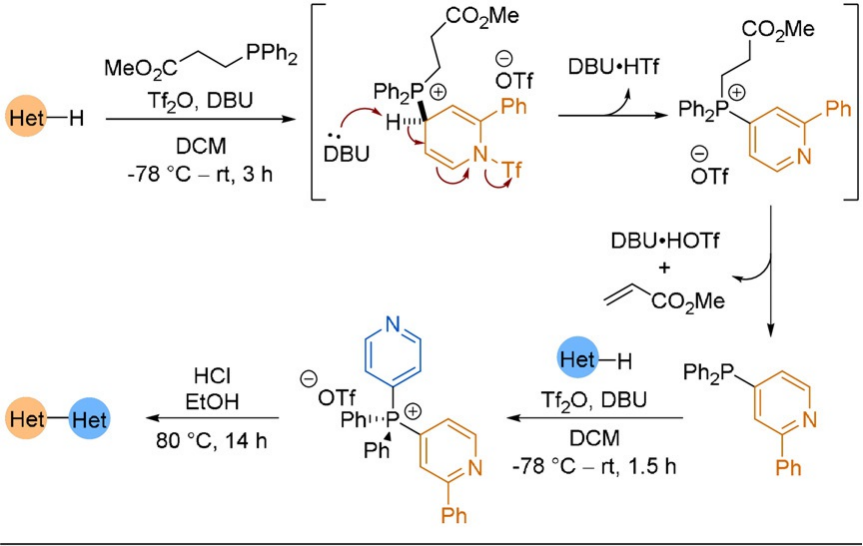
Heterobiaryl three‐step synthetic sequence via phosphorus ligand coupling. DBU=1,8‐diazabicyclo[5.4.0]undec‐7‐ene.

In 2018, McNally applied contractive phosphorus C−C cross‐coupling from C−H precursors to the 2‐pyridyl problem.^[128]^ The key step was the migration of one heterocycle to the *ipso* position of the second, around a central pentacoordinate P^V^ atom (Scheme 29). The reaction required a specific addition sequence of reagents to ensure the pyridine was activated for nucleophilic addition as well as correct phosphonium salt formation. While C−H precursors are considered an atom‐economical starting material, they often suffer from a lack of regioselectivity in reactions. However, when no substituents were present on the pyridine ring, the reaction was completely selective for the 4‐position, switching only to the 2‐position when the 4‐site was blocked. Functional groups, such as esters, trifluoromethyl groups and halides, were tolerated to give a range of unsymmetrical biheteroaryl products, including 2,2′‐bipyridines and complex drug molecules. However, other common functional groups performed poorly (alcohols, phenols and alkyl‐substituted amides) because of their tendency to react with the strong acids employed. Additionally, pyridines and diazines with more than two EWGs or EDGs reacted poorly.

**Scheme 30:**
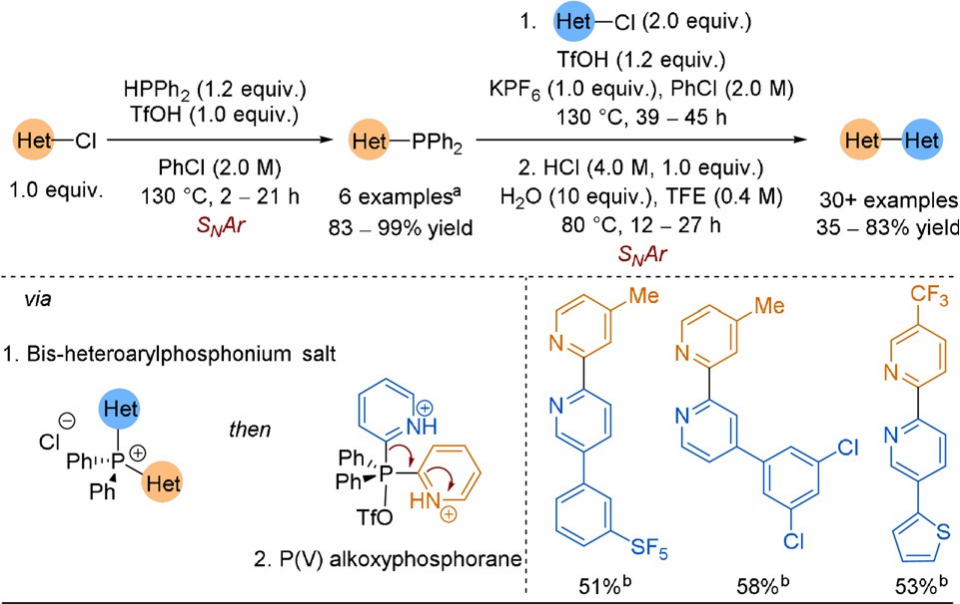
Scope examples of 2,2′‐bipyridines formed by P^V^ contractive methodology. [a] Alternative conditions were used for quinolines/diazines (6 examples, 62–95 % yield). [b] Yields after coupling (steps 1 and 2).

In order to further the utility of this P^V^ contractive coupling methodology to the 2‐pyridyl problem, McNally and co‐workers returned in 2019 with an advancement.^[129]^ Instead of the previous C−H functionalisation approach, chloroazines and heteroaryl phosphines were used as substrates. The latter were prepared in a single step from the corresponding heteroaryl chloride (Scheme 30). Once isolated, the heteroaryl phosphines could undergo S_N_Ar with a second heteroaryl chloride to generate a key bis‐heteroarylphosphonium salt intermediate. Strong acids were required to protonate the pyridine nitrogen atoms, forming a P^V^ alkoxyphosphorane intermediate.

The methodology was applied to a broad scope of biaryls. Improved regioselectivity from their 2018 work was demonstrated by the tolerance of C−H in the 4‐position of 2‐pyridine coupling partners. Unfortunately, key drawbacks are that the process requires strong acids and high reaction temperatures for many hours to result in mostly moderate yields of heterobiaryl products (Scheme 30). However, some scope examples would be challenging to obtain by classical organometallic chemistry. For example, comparing this P^V^ approach to Stille and Negishi couplings to molecules containing multiple halides showed the P^V^ route to be superior owing to its complete regioselectivity for the most S_N_Ar active halide. Orthogonality was explored, showing that the phosphine remained intact through a SMC. Most importantly, placing PPh_2_ at the 2‐pyridyl position gave a far higher yield of the 2,2′‐bipyridine than methods with either the BF_3_K salt or BMIDA, which had also been previously developed as solutions for the 2‐pyridyl problem.

**Scheme 31:**
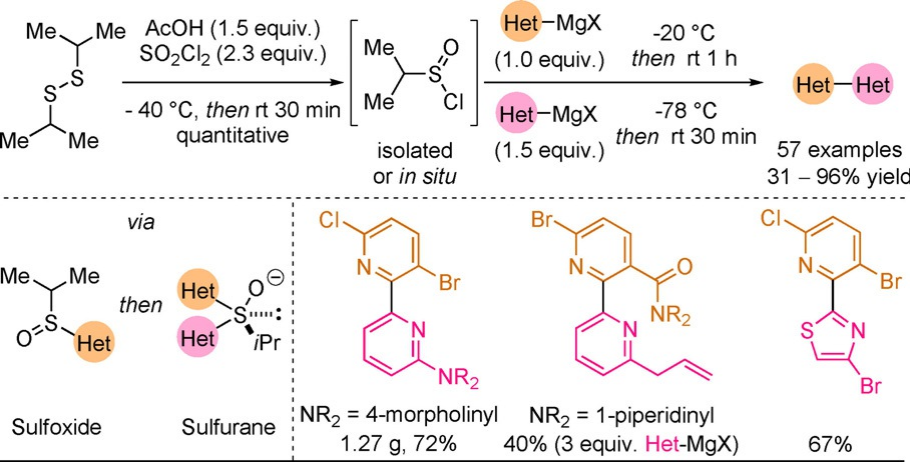
Sulfur(IV)‐mediated unsymmetrical heterocycle cross‐couplings with selected 2‐pyridyl scope examples.

In 2020, Qin and co‐workers reported that oxidative cross‐couplings of Grignard nucleophiles could be mediated by sulfinyl chlorides.^[130]^ The sequential assembly of two Grignard reagents leads to sulfuranes via sulfoxides (Scheme 31). Compared to the titanate work shown in Section 2.3, there is no need for added metal or oxygen to trigger the reductive elimination from the sulfurane complex. Although these sulfur(IV)‐based coupling methods have been known since the 1980s,^[14a]^ the novelty in the work of Qin and co‐workers is the use of isopropylsulfinyl(IV) chloride. This sulfur(IV) derivative could be conveniently prepared and stored at 4 °C for months without loss of reactivity, or could be generated in situ using Herrmann's protocol.^[131]^ A large scope of 2,2′‐linked diazines (>40 examples) was obtained in moderate to excellent yields (35–96 % yield), and a range of functionalities including halides, alkene, alkynes, acetals, esters, nitriles and amides were tolerated, owing to the high reaction rates and low temperatures needed for the coupling.

Utilisation of the chemistry of main group elements to perform cross‐coupling reactions, rather than relying on precious transition metals, could become a popular area for synthetic chemists to explore,^[132]^ especially as more sustainable and greener solutions are sought.

## 5 C−H Activation

**Scheme 32:**
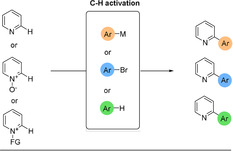
C−H activation to solve the 2‐pyridyl problem.

Three categories of C−H activation are considered, depending on the nature of the pyridine's coupling partner (Scheme 32): i) Double C−H activation or cross dehydrogenative coupling (CDC); ii) Coupling with an electrophile (mostly organohalides); iii) Coupling with a nucleophile (such as Grignard or boron‐based reagents).

C−H‐activated coupling reactions with nucleophiles^[133]^ will not be addressed here, as the focus is on processes in which the pyridine group is not the electrophilic partner. While CDC does not use the pyridine moiety as a nucleophile, it is also not used as an electrophile and is an interesting alternative solution to the 2‐pyridyl problem.^[134]^

This section is divided into 3 subsections, focusing first on the activation of free pyridines, then N‐oxides, and finally more recent developments of novel derivatives.

### 5.1 Pyridines

Direct C−H activation of pyridines is still an underdeveloped area, with only moderate success achieved so far, because of the need for harsh reaction conditions, limited scopes and/or poor selectivity.^[18b]^ As such, pyridines are often used as directing groups rather than reactive species in C−H activation processes.^[16a]^

#### 5.1.1 Cross Dehydrogenative Coupling (CDC)

Concerning direct pyridine homocoupling, the symmetrical 2,2′‐bipyridine motif has been achieved through various catalytic methods using Raney nickel,^[135]^ Pd/C,^[136]^ as well as ruthenium or tantalum complexes in stoichiometric^[137]^ then sub‐stoichiometric amounts.^[138]^ These methods had several limitations, such as narrow scopes, high temperatures, and modest yields.

**Scheme 33:**
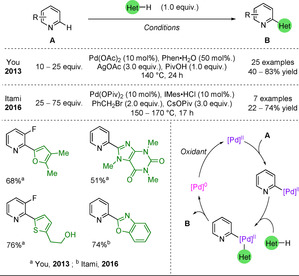
Pd‐catalysed CDC to heterobiaryl products.

In 2013, a palladium‐catalysed oxidative cross‐coupling was developed by You and co‐workers (Scheme 33).^[139]^ In this reaction, both reagents coordinate to the palladium and the resulting complex undergoes reductive elimination, forming the desired product. A stoichiometric quantity of oxidant, here a silver salt, is required to oxidise the Pd^0^, closing the catalytic cycle. The need for such a large excess of the pyridine, used as both reagent and solvent, remains the main limitation. Alternatively, a Rh^III^ catalyst was used by Su and co‐workers, but required the pyridine to carry an amide directing group to control the regioselectivity.^[140]^ In 2016, Itami and co‐workers coupled pyridines with benzoxazoles using an organohalide as the oxidant (Scheme 33).^[141]^

#### 5.1.2 Coupling with an Electrophile

**Scheme 34:**
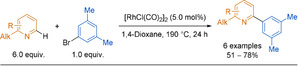
Rh‐catalysed C−H activation of pyridines with electrophiles.

In 2008, Ellmann and co‐workers used a Rh^I^ catalyst to form 2‐arylpyridines from pyridines and aryl bromides (Scheme 34).^[142]^ Substitution on the pyridine moiety was limited to simple alkyl chains and substitution vicinal to the nitrogen centre was needed in order to limit rhodium binding to the nitrogen. Excess of the pyridine reagent and high temperatures were the main limitations. Notably, using a palladium catalyst instead leads to 3‐arylation rather than the desired 2‐arylation.^[143]^

### 5.2 Pyridine N‐Oxides

Replacing pyridines by their N‐oxide derivatives has a significant number of advantages. The oxide component serves as both an activating and directing group, lowering the acidity and free‐energy barrier of the C−H bond on the N‐oxide compared to that of the corresponding pyridine,^[144]^ thus enhancing the reactivity and the regioselectivity to the desired 2‐position.^[145]^ Furthermore, pyridine N‐oxides are generally bench‐stable solids and commercially available (or easily accessible by pyridine oxidation). However, they require an extra reduction step to yield the 2‐arylpyridine moiety and often lead to significant amounts of the 2,6‐diarylated product.

#### 5.2.1 Cross Dehydrogenative Coupling

**Scheme 35:**
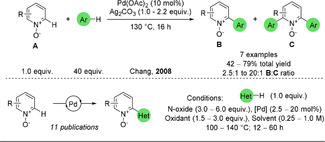
Pd‐catalysed CDC of pyridine N‐oxides.

In 2008, Chang and co‐workers developed a double C−H activation between pyridine N‐oxides and unactivated arenes (Scheme 35).^[146]^ The reaction follows a similar pathway as the CDC for pyridines (see Scheme 33). The unactivated arenes needed to be in large excess and were used as the solvent. A great many research groups have since developed variants of the same methodology, using activated heterocycles which circumvent the need for such an excess of substrate (Scheme 35).^[147]^

**Scheme 36:**
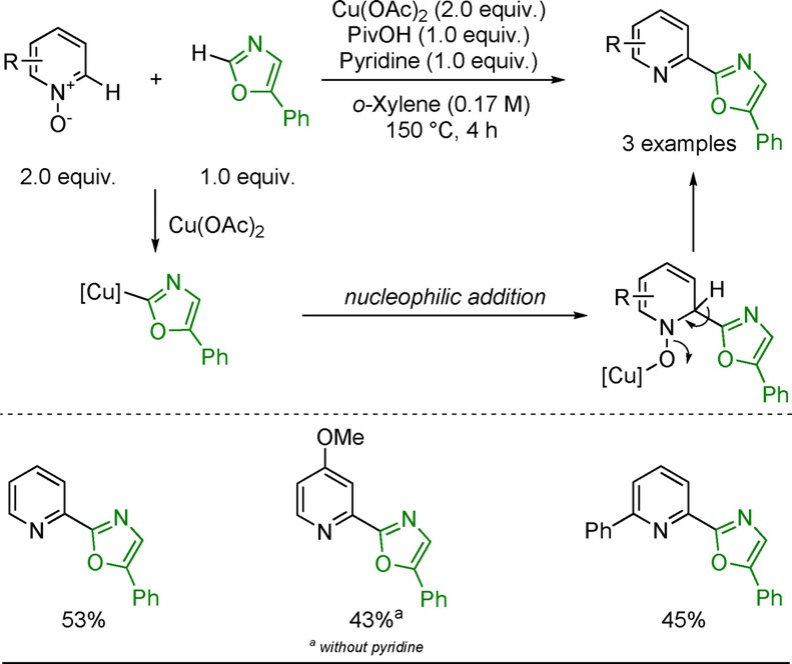
Cu‐mediated CDC of pyridine N‐oxides.

A copper‐assisted coupling between the N‐oxide and an oxazole was developed by Miura and co‐workers in 2015 (Scheme 36).^[148]^ The copper has a dual role, activating the oxazole to add onto the N‐oxide, then binding to the oxygen of the pyridine N‐oxide and allowing re‐aromatisation by deoxygenative elimination. While the scope is limited and the yields modest, this method allows for Pd‐free arylation of the pyridine N‐oxide in only 4 hours without needing a further reductive step. Pyridine was used as an additive, providing evidence of the unreactive nature of pyridines to this C−H activation.

**Scheme 37:**
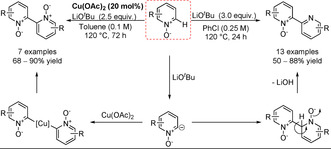
Alternative routes to pyridine N‐oxide C−H activation.

A Pd‐free homocoupling of pyridine N‐oxide was developed through the use of a strong base (Scheme 37).^[149]^ Depending on whether or not copper acetate was added, the reaction would go through two different pathways: S_N_Ar (although it might be a radical addition^[150]^) or a copper‐catalysed oxidative coupling. While both pathways enable the homocoupling, the S_N_Ar pathway's final deoxygenative rearomatisation step produces an N‐oxide rather than the N,N′‐dioxide.

#### 5.2.2 Coupling with an Electrophile

**Scheme 38:**
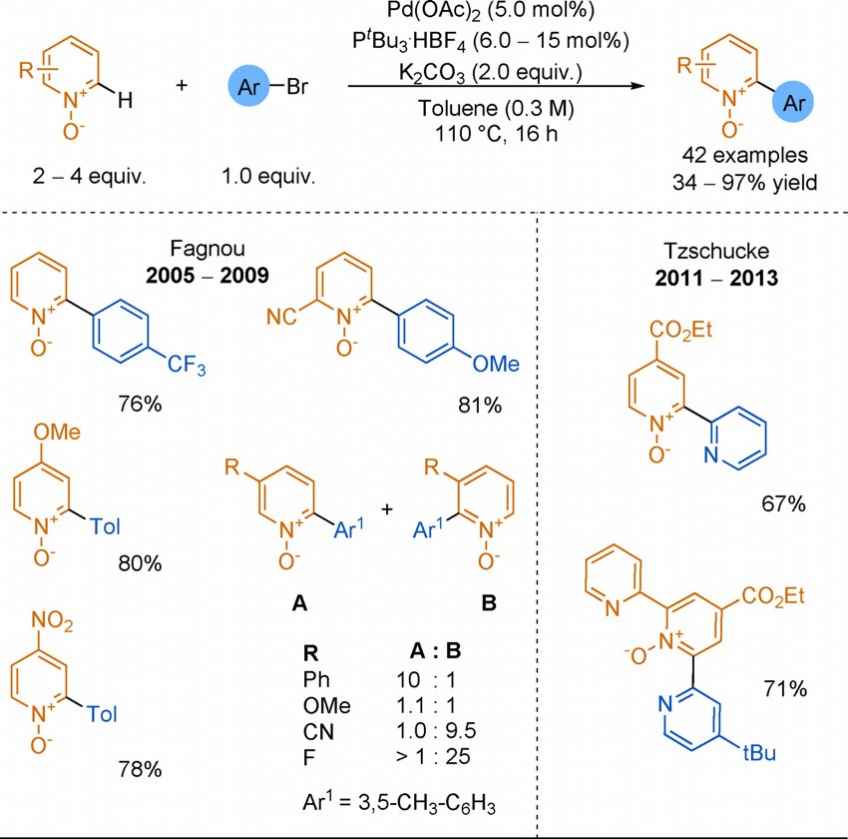
Pd‐catalysed C−H activation of pyridine N‐oxides with electrophiles.

In 2005, Fagnou and co‐workers published the first example of C−H activation of pyridine N‐oxides by developing a palladium‐catalysed cross‐coupling between an aryl bromide and an N‐oxide, with a broad scope, high yields, and complete regioselectivity at the 2‐position (Scheme 38).^[151]^ Steric and electronic effects did not significantly impact the yield and a further Pd/C reduction allowed access to the desired 2‐arylpyridines. This synthetic methodology was extended to a broad scope of various N‐oxides and coupling partners.^[152]^ Regarding pyridine N‐oxides, substitution at any position on the ring had little impact on yield, but regioselectivity issues appeared when the ring was substituted at the 3‐ or 5‐positions. Tzschucke and co‐workers used these reaction conditions with 2‐bromopyridines to form unsymmetrical bipyridines^[153]^ and terpyridines (Scheme 38).^[154]^

**Scheme 39:**
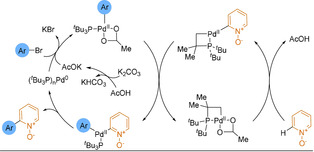
Catalytic cycle for the C−H activation of pyridine N‐oxides with electrophiles.

The mechanism was studied by the groups of Fagnou^[155]^ and Hartwig.^[156]^ It proceeds through a bimetallic palladium catalytic cycle, in which the aryl halide and the pyridine N‐oxide each bind onto a different palladium complex. Transmetalation and reductive elimination give the desired product, closing the catalytic cycle. The presence (and regeneration) of acetate and tris(*tert*‐butyl)phosphine are essential to the catalytic cycle, as both bind to palladium to form reactive species (Scheme 39).

**Scheme 40:**
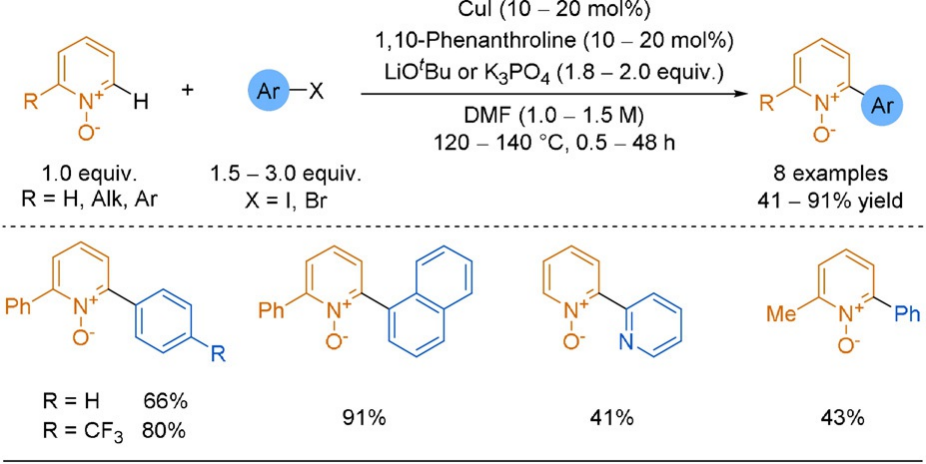
Cu‐catalysed C−H activation of pyridines.

An alternative C−H activation of pyridine N‐oxides was developed by the groups of Daugulis and You, replacing the palladium catalyst with a cheaper copper catalyst (Scheme 40).^[157]^ When the 6‐position was not blocked, diarylation could be observed.

### 5.3 Pyridinium Derivatives Coupled with an Electrophile

**Scheme 41:**
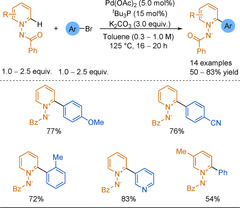
Coupling N‐iminopyridinium ylides to electrophiles.

Building on Fagnou's work, in 2008 Charette and co‐workers replaced the pyridine N‐oxide with a N‐iminopyridinium ylide (Scheme 41).^[158]^ As the amide functionality on the ylide is a stronger Lewis base—therefore a better directing group—than the N‐oxide, this allows for an easier C−H insertion. With only a small excess of the ylide (1.5 vs. 4.0 equiv for the N‐oxide), the arylation was performed on a broad scope with good yields. However, obtaining the N‐functionalised pyridine requires two extra steps (methylation then reduction), rather than one in the case of the N‐oxide. Contrary to pyridine N‐oxides, only the unsubstituted ylide is commercially available and all others require synthesis.

**Scheme 42:**
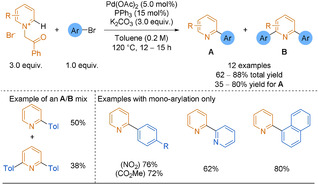
Coupling N‐phenacylpyridinium halides with electrophiles.

The next year Wang, Hu and co‐workers also modified the activating group, using N‐phenacylpyridinium halides (Scheme 42).^[159]^ The activating group on the pyridine is cleaved at the end of the reaction through enolisation, therefore no supplementary deprotection steps are necessary to obtain the 2‐arylpyridine. However, a lack of selectivity between the mono‐ and diarylated products was often observed.

**Scheme 43:**
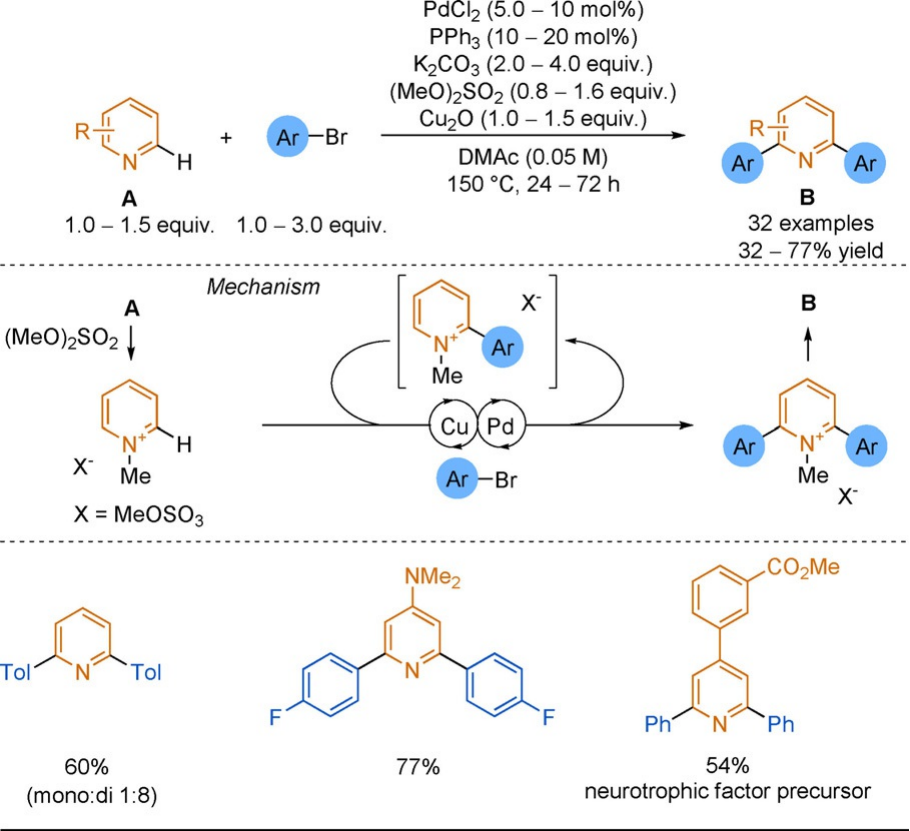
Coupling in situ generated N‐methyl pyridinium salts with electrophiles.

Finally, Chen and co‐workers developed a variation on the method, involving the use of a traceless activating group (Scheme 43).^[160]^ The pyridine undergoes in situ N‐methylation to produce the methyl pyridinium, followed by copper‐assisted palladium‐catalysed C−H activation and subsequent demethylation, yielding the desired diarylpyridines. The diarylation is favoured over monoarylation by design rather than default yet unsymmetrical 2,6‐disubstituted pyridines can still be obtained by prefunctionalising one of the positions.^[161]^ Similar reaction conditions have also been applied to 2‐picolinic acid derivatives for the synthesis of 2,6‐diarylpyridines.^[162]^

## 6 Conclusion

The landscape of 2‐pyridyl nucleophiles was initially dominated by both tin and zinc reagents. The former approach is now less commonly employed in arylation reactions due to toxicity issues, but the latter has seen considerable improvement with the advances in palladium catalysis and the development of highly efficient ligand systems. Such progress has also enabled the use of other 2‐pyridyl nucleophiles, namely silanes, Grignard reagents, germanes, alanes and indanes.

Traditionally the coupling of 2‐pyridyl boronic acids was plagued with poor reaction success owing to their instability. However, the popularity of SMC has led to considerable development of more efficient catalytic systems and new 2‐pyridyl boron nucleophiles. Innovative strategies, from stabilised, slow‐release boronates to Lewis acid additives, have transformed the efficacy of 2‐pyridyl boron nucleophiles in SMC reactions. The promising recent success of stable amino boronates (Bdan) shows there is still momentum in the quest for stable, yet reactive 2‐pyridyl boron nucleophiles.

More recently, alternative, novel nucleophiles have emerged as excellent solutions to the 2‐pyridyl problem, such as sulfinate salts. While decarboxylation strategies show promise, in order to truly harness the abundance of green 2‐pyridyl carboxylate starting materials, further work is needed to elude undesirable side reactions.

Finally, direct C−H activation also offers a solution to the 2‐pyridyl problem. The pyridines’ poor results towards C−H activation can be remedied by using the corresponding N‐oxides and related derivatives; however, their use requires added synthetic steps and can lead to over‐arylation. Identifying a balance between reagent accessibility and significant, yet selective reactivity remains the main challenge of pyridine C−H activation.

It is important to note that the synthesis of 2‐pyridyl nucleophiles is often limited. Many approaches require pyridyl nucleophiles to be synthesised via lithiation (e.g. alanes, germanes, boronates and traditional organometallic reagents), which in turn constrains functional group tolerance on the reagent. Some modern methodologies, such as desulfinative, decarboxylative, P^V^ contractive and organozirconium couplings, are not restricted in this way. Future efforts to improve efficacy, sustainability and functional group tolerance of both nucleophile synthesis and cross‐coupling processes, will be necessary for increasing industrial application.

With these tools in hand, industrially important 2‐pyridyl(hetero)aryl frameworks are now more accessible than ever. We hope that the inventive strategies discussed herein should provide a resource for both the 2‐pyridyl problem and the coupling of other challenging heteroaromatic substrates. The development of novel nucleophiles and cross‐coupling conditions will continue and we hope that the chemistry explored in this Review proves a useful tool for future innovation.

## Conflict of interest

The authors declare no conflict of interest.

## Biographical Information

*Xinlan Cook received her MChem degree from the University of York in 2017, completing her master's thesis during a year‐long placement at F. Hoffmann‐La Roche (Basel, Switzerland), synthesising novel molecules for the treatment of Alzheimer's disease. She is now completing her doctoral research in the group of Prof. M. C. Willis at the University of Oxford, investigating novel reagents for C(sp^2^)−C(sp^2^) desulfinative cross‐coupling reactions*.

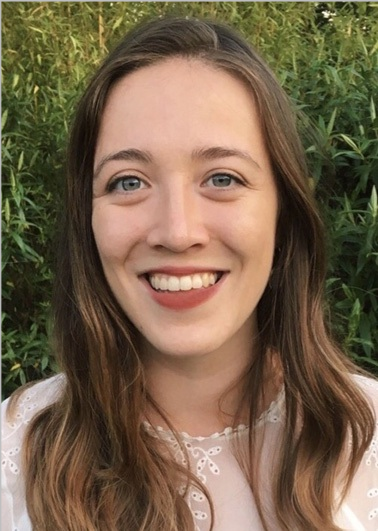

## Biographical Information

*Antoine de Gombert graduated from Chimie ParisTech and Université Pierre et Marie Curie with a Diplôme d'Ingénieur and MSci. After working on visible light catalysis at CSIRO (Australia), he completed a 6‐month internship at F. Hoffmann‐La Roche in Switzerland. He is currently a PhD student with Prof. M. C. Willis at the University of Oxford, where his research focuses on the mechanistic aspects of palladium‐catalysed desulfinative cross‐coupling reactions*.

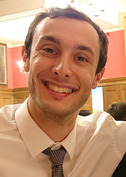

## Biographical Information

*Janette McKnight obtained her MChem degree from the University of Bath in 2017. During this time, she undertook an industrial placement year in process chemistry at GSK Stevenage, before returning to complete a final year project in the field of C−H activation with Prof. C. Frost. She moved to the University of Oxford for postgraduate study, where she is currently working on C(sp^2^)−C(sp^3^) desulfinative cross‐coupling reactions under the supervision of Prof. M. C. Willis*.

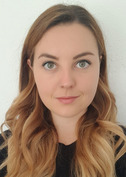

## Biographical Information

*Loïc Pantaine obtained his PhD in 2016, concerning asymmetric aminocatalysis, with Prof. C. Greck and Dr. V. Coeffard (Université Paris‐Saclay). In 2017, he joined the teams of Dr. G. Masson (ICSN) and Dr. C. Bour (Université Paris‐Sud) for his first postdoctoral fellowship on photoredox/gold dual catalysis. In 2018, he worked on photoredox catalysis in the group of Prof G. A. Molander (University of Pennsylvania) and is currently working with Prof M. C. Willis (University of Oxford) on desulfinative cross‐coupling reactions*.

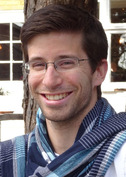

## Biographical Information

*Michael Willis received his undergraduate education at Imperial College London and his PhD from the University of Cambridge working with Prof. S. V. Ley, FRS. After a postdoctoral stay with Prof. D. A. Evans at Harvard University, he was appointed to a lectureship at the University of Bath in November 1997. In January 2007 he moved to the University of Oxford, where he is a now a Professor of Chemistry and a Fellow of Lincoln College. His group's research interests are based on the development and application of new catalytic processes for organic synthesis*.